# Crystal Structure of the Hendra Virus Attachment G Glycoprotein Bound to a Potent Cross-Reactive Neutralizing Human Monoclonal Antibody

**DOI:** 10.1371/journal.ppat.1003684

**Published:** 2013-10-10

**Authors:** Kai Xu, Barry Rockx, Yihu Xie, Blair L. DeBuysscher, Deborah L. Fusco, Zhongyu Zhu, Yee-Peng Chan, Yan Xu, Truong Luu, Regina Z. Cer, Heinz Feldmann, Vishwesh Mokashi, Dimiter S. Dimitrov, Kimberly A. Bishop-Lilly, Christopher C. Broder, Dimitar B. Nikolov

**Affiliations:** 1 Structural Biology Program, Memorial Sloan Kettering Cancer Center, New York, New York, United States of America; 2 Sealy Center for Vaccine Development, Departments of Pathology and Microbiology and Immunology, University of Texas Medical Branch, Galveston, Texas, United States of America; 3 Laboratory of Virology, National Institutes of Health, Rocky Mountain Laboratories, Hamilton, Montana, United States of America; 4 Division of Biological Sciences and the University of Montana, Missoula, Montana, United States of America; 5 Department of Microbiology and Immunology, Uniformed Services University, Bethesda, Maryland, United States of America; 6 Protein Interactions Group, CCRNP, CCR, Frederick National Laboratory for Cancer Research, National Institutes of Health, Frederick, Maryland, United States of America; 7 Naval Medical Research Center, NMRC-Frederick, Fort Detrick, Maryland, United States of America; 8 Henry M. Jackson Foundation, Bethesda, Maryland, United States of America; 9 Department of Medical Microbiology, University of Manitoba, Winnipeg, Manitoba, Canada; Institut Pasteur, France

## Abstract

The henipaviruses, represented by Hendra (HeV) and Nipah (NiV) viruses are highly pathogenic zoonotic paramyxoviruses with uniquely broad host tropisms responsible for repeated outbreaks in Australia, Southeast Asia, India and Bangladesh. The high morbidity and mortality rates associated with infection and lack of licensed antiviral therapies make the henipaviruses a potential biological threat to humans and livestock. Henipavirus entry is initiated by the attachment of the G envelope glycoprotein to host cell membrane receptors. Previously, henipavirus-neutralizing human monoclonal antibodies (hmAb) have been isolated using the HeV-G glycoprotein and a human naïve antibody library. One cross-reactive and receptor-blocking hmAb (m102.4) was recently demonstrated to be an effective post-exposure therapy in two animal models of NiV and HeV infection, has been used in several people on a compassionate use basis, and is currently in development for use in humans. Here, we report the crystal structure of the complex of HeV-G with m102.3, an m102.4 derivative, and describe NiV and HeV escape mutants. This structure provides detailed insight into the mechanism of HeV and NiV neutralization by m102.4, and serves as a blueprint for further optimization of m102.4 as a therapeutic agent and for the development of entry inhibitors and vaccines.

## Author Summary

Since their initial emergence, henipaviruses have continued to cause spillover events in both human and livestock populations, posing significant biothreats. Currently there are no licensed or approved therapies for treatment of henipavirus infection and the human case mortality rates average >70%. We used X-ray crystallography to determine the high-resolution structures of the Hendra virus G glycoprotein in complex with a cross-reactive neutralizing human monoclonal antibody. The structures provide detailed insight into the mechanism of HeV and NiV neutralization by this potent and clinically-relevant human monoclonal antibody that is currently in development for use in humans. This monoclonal antibody was recently shown to be an effective post-exposure therapy in non-human models of lethal Hendra virus infection. Indeed, it has already been used in four people on a compassionate use request, three in Australia and one in the United States, as a therapeutic agent. Furthermore, we identified and characterized two escape mutants generated *in vitro* and evaluated their mechanism of escape. Our results serve as a blueprint for further optimization of this antibody and for the development of novel entry inhibitors and vaccines. This report also supports the additional pre-clinical data required for eventual licensure by detailing the antibody's mechanism of henipavirus neutralization.

## Introduction

Henipaviruses, Hendra virus (HeV) and Nipah virus (NiV) [1], are recently emerged, highly pathogenic paramyxovirus zoonoses whose major reservoirs in nature are several species of pteropid fruit bats [2], [3]. HeV causes lethal respiratory disease and encephalitis in horses and severe respiratory disease or late onset encephalitis in humans. In total, there have now been 39 HeV spillover events in Australia including 7 cases of human infection with 4 fatalities since 1994 [4]–[9]. NiV subsequently emerged in peninsular Malaysia in 1998–99, causing a large outbreak of respiratory disease in pigs and encephalitis among pig farmers, and was later shown to be closely related to HeV [1]. Similar to HeV, nearly annual outbreaks of NiV infection have been observed. These NiV outbreaks have been associated with significantly higher case fatality rates in people, up to 100%, and several outbreaks have also been linked to the consumption of raw date palm sap contaminated with virus as well as human-to-human transmission [10]–[12]. To date, there have been 570 reported cases of NiV infection in people with 305 fatalities [8], [13], [14]. The unusual broad species tropism, high morbidity and mortality rates, as well as the lack of any licensed therapeutics, have rendered the henipaviruses Biological Safety Level-4 (BSL-4) pathogens and potential biological threats to humans and livestock.

An often utilized approach to antivirus drug design is to block viral entry via small molecules, peptides and neutralizing monoclonal antibodies (mAbs) that bind to the viral surface glycoproteins. A unique feature of the majority of paramyxoviruses is that they require two surface glycoproteins for host cell entry: a Class I fusion (F) glycoprotein and an attachment glycoprotein, which can be a hemagglutinin–neuraminidase (HN), hemagglutinin (H), or as in the case for henipaviruses a G glycoprotein that has neither hemagglutinating nor neuraminidase activities [2]. The henipavirus G glycoprotein engages the host cell membrane protein receptors ephrin-B2 and -B3, and this initial interaction is believed to be sufficient to trigger the F-mediated fusion event between the viral envelope and the host cell membrane leading to virus entry [15], [16], [17]. In the absence of available vaccines or antiviral drugs, neutralizing hmAbs offer the possibility for effective pre- and/or post-exposure treatment for many important human viral infections. Previously, several hmAbs, m101–m107, were isolated using a recombinant soluble Hendra virus G (HeV-G) glycoprotein as the antigen for panning of a large naïve antibody library [18]. Among the hmAbs, m102 and its derivatives (m102.1-8) generated by heavy chain random mutations and light chain shuffling, showed improved binding to HeV-G; clone m102.4 had equal or higher binding affinity than the other clones and was selected for further characterization and converted to an IgG1 format [19]. The m102.4 hmAb was able to cross-react with both NiV-G and HeV-G *in vitro* with 50% inhibitory concentrations (IC_50_)of less than 40 ng/ml and 600 ng/ml for Nipah virus and Hendra virus, respectively, and is capable of neutralizing all available isolates of HeV and NiV [19], [20]. In animal disease models, m102.4 has been shown capable of protecting ferrets against a lethal NiV challenge [20], as well as African green monkeys (AGM) against a lethal HeV challenge [21], [22], in time frames of 10 to even 72 hours post viral exposure, respectively. In light of the experimental success of this post-exposure treatment of both NiV and HeV infection, m102.4 has since been administered on a compassionate use basis to two individuals in Australia with a high risk of HeV exposure during the 2010 HeV spillover, and again in 2012 in another person exposed to HeV. In 2013, m102.4 was used again by compassionate use protocol in an individual with a laboratory exposure to NiV in United States. In all these cases, none of these individuals showed symptoms of HeV or NiV infection at the time of m102.4 administration, and all individuals remain in good health to date. Altogether, as a fully human mAb, m102.4 shows promise as a potential prophylactic or therapeutic agent against henipavirus infection, and appears to be suitable for controlled safety trials in humans.

To fully characterize the binding epitope [19], as well as the binding and recognition mechanism, we determined and here present the crystal structure of the complex between the globular head domain of HeV-G and the Fab domain of m102.3, a close derivative of m102.4, featuring an identical heavy chain and a similar light chain. The structure reveals the molecular mechanism of neutralization and cross-reactivity and provides a basis for further improvement of m102.4, including efficacy enhancement and escape mutant prevention. Additionally, the presented structural information may aid the development of a henipavirus vaccine or other specific entry inhibitors.

## Results

### Structure of the m102.3/HeV-G protein complex

The head domain of HeV-G (residues 171-602) was produced using the baculovirus expression system, and the Fab domain of m102.3 was expressed in HB2151 cells. The protein complex was generated by mixing HeVsG with hmAb in a 1∶1.5 molar ratio followed by a 6 hour incubation and purification by Size-Exclusion Chromatography (SEC). The peak fractions containing the complex were collected and used for crystallization.

We obtained two crystal forms of the m102.3/HeV-G complex and used molecular replacement to determine the structures at 2.7 Å resolution (in space group P6_1_22) and at 2.8 Å resolution (in space group I222) (Table S1). There is one 1∶1 complex per asymmetric unit in both crystal forms. The overall structures in the two crystal forms are very similar (Figure S1) and the region containing the HeV-G molecules and the complementarity determining region-3 of the heavy chain (CDR-H3) of the Fabs can be superimposed with an r.m.s.d of 0.3 Å for 386 Cα atoms, while the two Fab structures, excluding just CDR-H3, can be superimposed with an r.m.s.d of 0.6 Å for 185 Cα atoms. The difference in the two structures is in the angle between CDR-H3 and the rest of the Fab, which consequentially causes a slight difference in the buried m102.3/HeV-G interface area: 1070 Å^2^ in crystal form I222, and 1010 Å^2^ in crystal form P6_1_22. However, most interface residues in the core region are the same in both crystal forms. For the remainder of this report and figures, we use the P6_1_22 structure.

**Figure 1:**
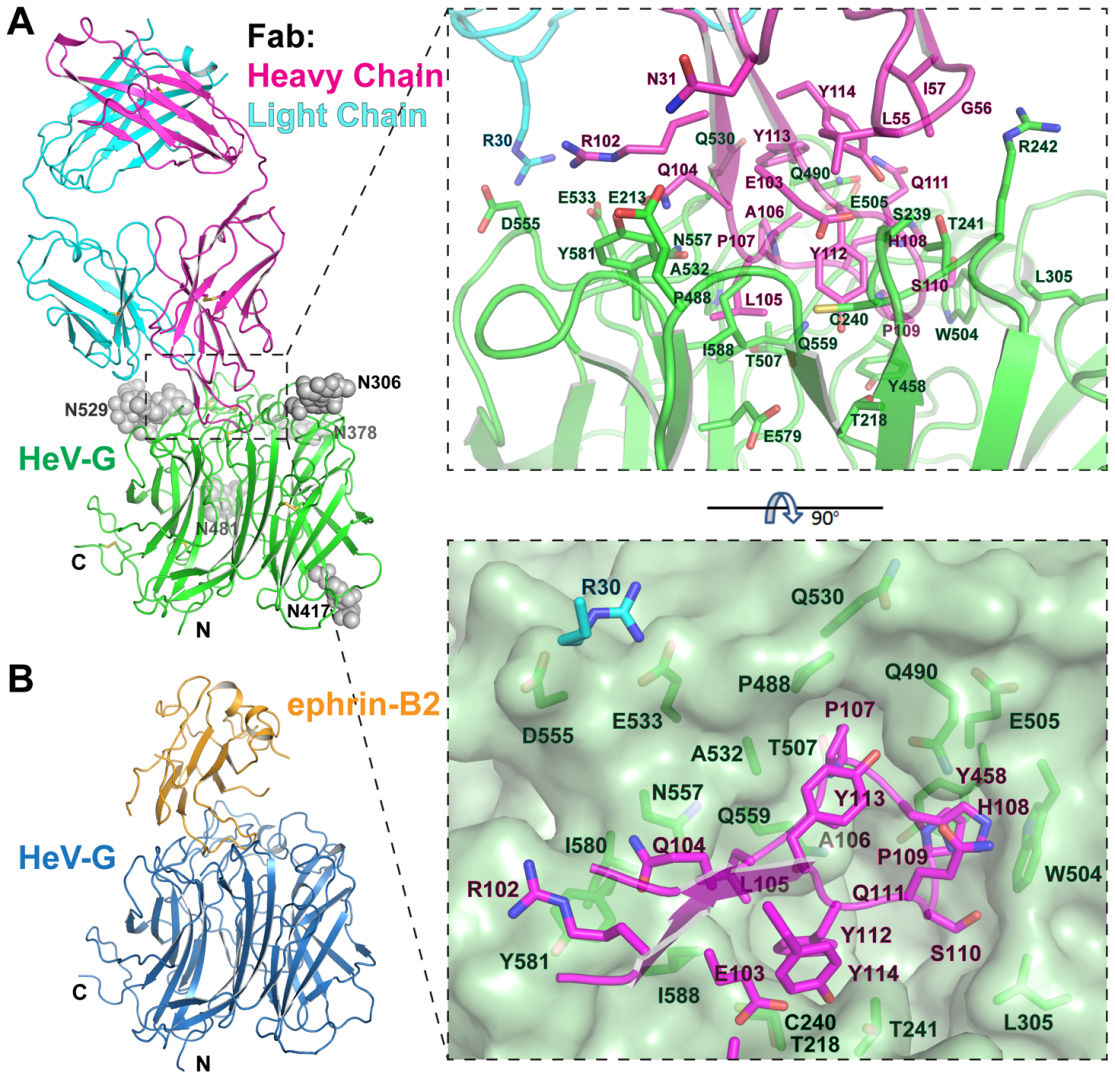
Structure of the m102.3/HeV-G complex, and comparison to the ephrin-B2/HeV-G structure. A. Left: Overall Structure of the m102.3/HeV-G complex viewed from the side. CDR-H3 (magenta) of m102.3 inserts into the central cavity of HeV-G (green). Disulfide bonds are shown as yellow sticks. The five glycosylation sites of HeV-G are shown as grey spheres. Right: A close up view of the HeVG/m102.3 complex interface. Residues involved in the interaction are shown as stick figures and labeled. The solvent accessible surface of HeV-G central cavity region, viewed from top, is presented on the bottom. CDR-H3 residues (magenta) and R30 (cyan, on the light chain of Fab) and their contacting residues on HeV-G (green) are shown and labeled. B. Overall structure of the ephrin-B2 (orange)/HeV-G (blue) complex. HeV-G in the complex is in the same orientation as in panel A.

**Figure 2:**
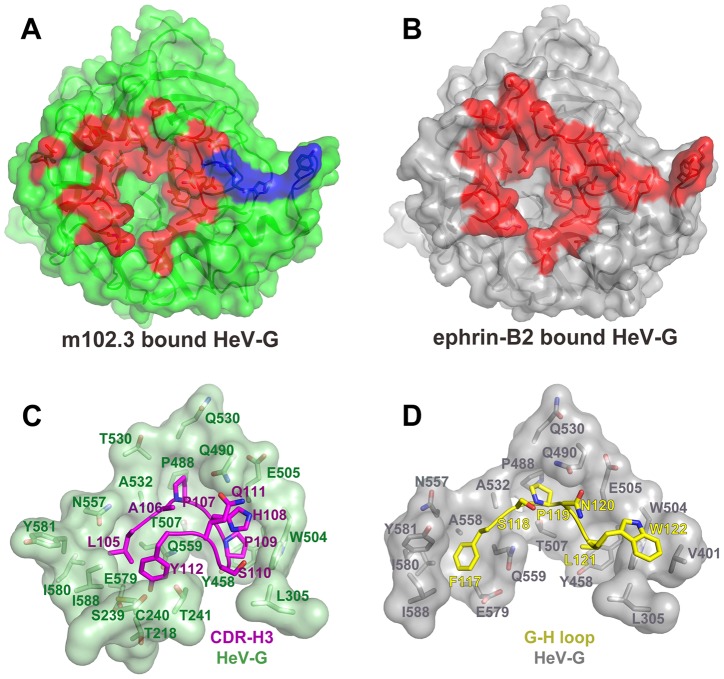
Comparison of the binding interfaces in the HeV-G/m102.3 and the HeV-G/ephrin-B2 complexes. A: The solvent accessible surface of the HeV-G molecule in the HeV-G/m102.3 complex viewed from the top. HeV-G is colored in green, except for the m102.3-contacting region that is colored in red (for the 1∶1 complex interface) and in blue (for the region contacted by another copy of the light chain in the hetero-tetrameric 2∶2 complex interface). B: The solvent accessible surface view of the HeV-G molecule in the HeV-G/ephrin-B2 complex viewed from the top. HeV-G is colored in grey, except for the ephrin-B2 contacting region, which is colored in red. C: The tip of the m102.3 CDR-H3 region (in magenta) bound in the HeV-G surface cavity (in green). D: The tip of the ephrin-B2 G-H loop (in yellow) bound in the HeV-G surface cavity (in grey).

The Fab binds a similar area on HeV-G (Figure 1A) as ephrin-B2, which is composed of a hydrophobic central cavity and a hydrophilic rim. Interestingly, CDR-H3 of the Fab approaches the HeV-G central cavity in a similar angle and from the same direction as the G-H loop of ephrin-B2 (Figure 1B). The interface involves Fab residues mostly located on CDR-H3, as well as three CDR-H2 residues (L55, G56 and I57), one CDR-H1 residue (N31) and one CDR-L1 residue (R30) (Figure 1). The long protruding CDR-H3 (23 residues) adopts a β-hairpin conformation, including a stalk and a tip (Q105-Y112). The central hydrophobic HeV-G/Fab contacts are formed by embedding the tip of CDR-H3 into the hydrophobic HeV-G receptor-binding cavity. A simulated annealing omit electron density map of this region is illustrated in Figure S2. Among the eight residues on the tip of CDR-H3, L105 is surrounded by Q559, A532, N557, Y581, I580, I588 and E579 of HeV-G; P107 is surrounded by T530, A532, P488, T507, Q490 of HeV-G; P109 is surrounded by E505, W504, Y458 of HeV-G; S110 is surrounded by L305 and W504 of HeV-G; and Y122 is surrounded by T241, C240, T218, S239 and E579 of HeV-G (Figure 1A and 2C). In addition, the side chain of Q111, H108 and P109 of the Fab are stacked together, with Q111, which forms the top layer of the stack and hydrogen bonds to Q490 of HeV-G. The surrounding hydrophilic HeV-G/Fab contacts involve residues on CDR-H3, CDR-L1 and CDR-H2. Notably, R30 on CDR-L1 forms salt-bridges with E533 and D555 of HeV-G; Hydrogen bonds are also formed between N31 on CDR-H1 and E213 of HeV-G, as well as between R102, E103, Y112, Y113 and Y114 on CDR-H3 and Y581, S239, T218, Q490 and T241 of HeV-G, respectively (Figure 1A). All HeV-G residues engaged by m102.3 are listed in Figure S7. Interestingly, Fab binding does not induce any significant conformational changes in HeV-G and the r.m.s.d in Cα positions between unbound and Fab-bound HeV-G is 0.257 Å. Some previously identified HeV-G mutations that were reported to affect the m102 or m102.4 binding [18], [19] are not part of the m102.3 epitope (Figure S3) suggesting that those mutations might affect the overall HeV-G structure.

### Hetero-tetrameric packing of the m102.3/HeV-G complex

The HeV-G head domain and Fab are both strictly monomeric, but, interestingly, when these two proteins are mixed together, they first form a hetero-dimeric complex that oligomerizes further in solution. Indeed the SEC assays indicate that 72 hours after mixing more than 90% of the complex migrates at a position corresponding to twice its original size in the gel-filtration column. An explanation of this phenomenon is provided by the crystal packing of the complex, where two copies of Fab and HeV-G assemble into a heterotetramer (Figure S4). It should be noted that the same 2∶2 heterotetrameric Fab/HeV-G complex assembly is observed in both crystal forms. As illustrated in Figure S2, the heterotetrameric m102.3/HeV-G assembly is generated by a two-fold crystallographic symmetry axis in which the two Fab molecules contact each other burying approximately 770 Å^2^ surface area on each side, while the two HeV-G molecules remain separate. Importantly, there is an additional contact area between the Fab light chains elbow region and the HeV-G molecule of the interacting complex, in which a further ∼240 Å^2^ are buried in each binding partner (Figure 2A, blue region). Thus, a total 1015 Å^2^ surface area is occluded on each side of the interface between the two 1∶1 complexes. Within the 2∶2 heterotetrameric complex, m102.3 and HeV-G form a more extensive interacting interface, rendering a more stable assembly. As shown in Figs. 2A and 2B, the total HeV-G surface region involved in the HeV-G/m102.3 interaction is almost the same as the one in the HeV-G/ephrin-B2, B3 interaction. However, the physiological relevance of this tetrameric assembly, and resulting cross-linking of mAb/G complexes on the viral membrane, needs to be studied further.

### Comparison of the HeV-G/m102.3 and HeV-G/ephrin-B2 complexes

Ephrin-B2 and m102.3 both interact with the receptor-binding surface of HeV-G, which includes a central hydrophobic cavity and surrounding hydrophilic rim. CDR-H3 of m102.3 resembles the G-H loop of ephrin-B2 in both its shape and the insertion angle into the HeV-G cavity (Figure 1). Most of the G-H loop-contacting residues of HeV-G also participate in the CDR-H3 binding (Figure 2 C, D). Unlike ephrin, the binding of m102.3 does not cause any significant conformational changes in HeV-G. Interestingly, although the tips of the G-H loop and CDR-H3 both target the pockets in the HeV-G central cavity, each of them uses slightly different residues and anchoring strategies. As shown in Figure 2D, from left to right, F117, P119, L121 and W122 on the ephrin G-H loop insert into four hydrophobic HeV-G pockets, occupying half of the HeV-G cavity; while L105, P107 and P109 on the m102.3 CDR-H3 insert into the first three of these same pockets (Figure 2C). Among these, ephrin P119 and P107 of CDR-H3 are strikingly similar. Additionally, the insertion of CDR-H3 further embeds S110 and Y112 into the other half of the HeV-G cavity. The side chain of H108 of CDR-H3 extends in the same direction as W122 of the G-H loop but does not reach the fourth pocket. Instead, it is embedded in a groove defined by Q490, W504, and E505 of HeV-G. A hydrogen bond between Q111 of CDR-H3 and Q490 of HeV-G further locks the H108 in this position, preventing the withdrawal of CDR-H3 from the binding cavity. Since the S110 contacting resides on HeV-G are L305 and W504, mutation of S110 to A or V could presumably enhance the interaction between m102.3 and HeV-G. In summary, CDR-H3 binds to HeV-G utilizing a very high affinity lock-and-key mode without inducing conformational changes in HeV-G.

### Comparison of m102.3 binding to NiV-G and HeV-G

**Figure 3:**
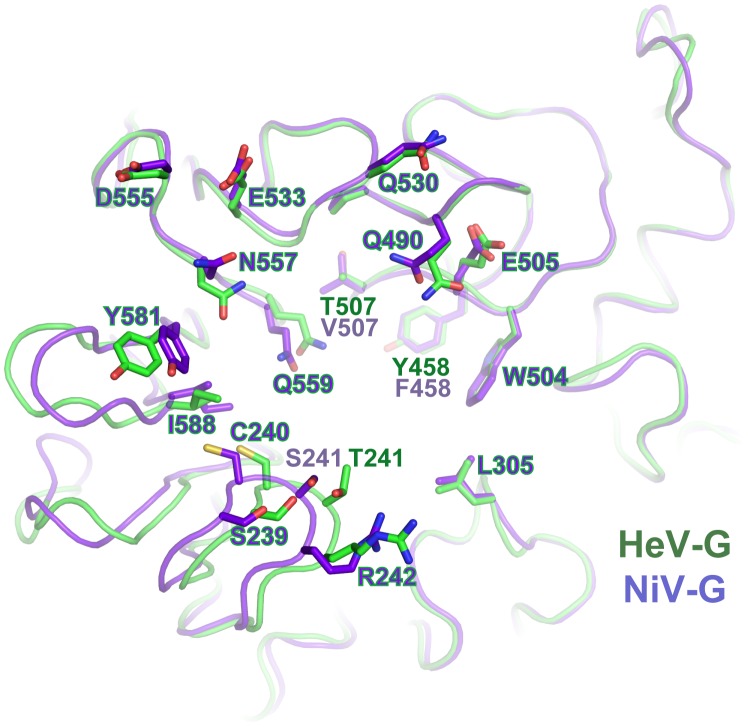
Comparison of the m102.3-binding regions of HeV-G and NiV-G. The HeV-G (green) and NiV-G (purple) structures are superimposed and viewed from top. The m102.3 contacting residues of HeV-G and the corresponding residues of NiV-G are shown and labeled. Most residues are conserved between HeV-G and NiV-G except for three: T/S241, T/V507 and Y/F458.

Although m102 mAb was originally isolated against HeV-G, it demonstrated a more potent neutralization capacity against NiV than HeV. Indeed, in vitro binding measurements using BioLayer Infetrometry (Table S2) document that both m102.3 and m102.4 display higher binding affinities for NiV-G than for HeV-G. Due to the high similarity between NiV-G and HeV-G, presumably m102.3 binds to NiV-G and HeV-G in a very similar manner, but an examination of the structure highlights some small structural differences that may explain the increased affinity of m102 for NiV-G. Upon superimposition of NiV-G to the m102.3-bound HeV-G, we found that only three residues at the mAb contacting regions are different. Among them, T507 and Y458 in HeV-G, which are part of the binding pockets for P107 and P109 of CDR-H3, are replaced by two hydrophobic residues, Valine and Phenylalanine, in NiV-G (Figure 3). Increasing hydrophobicity in this area very likely strengthens the predominantly hydrophobic interaction between the G protein and m102.3, enhancing its neutralizing activity.

### m102.3 vs. m102.4

**Figure 4:**
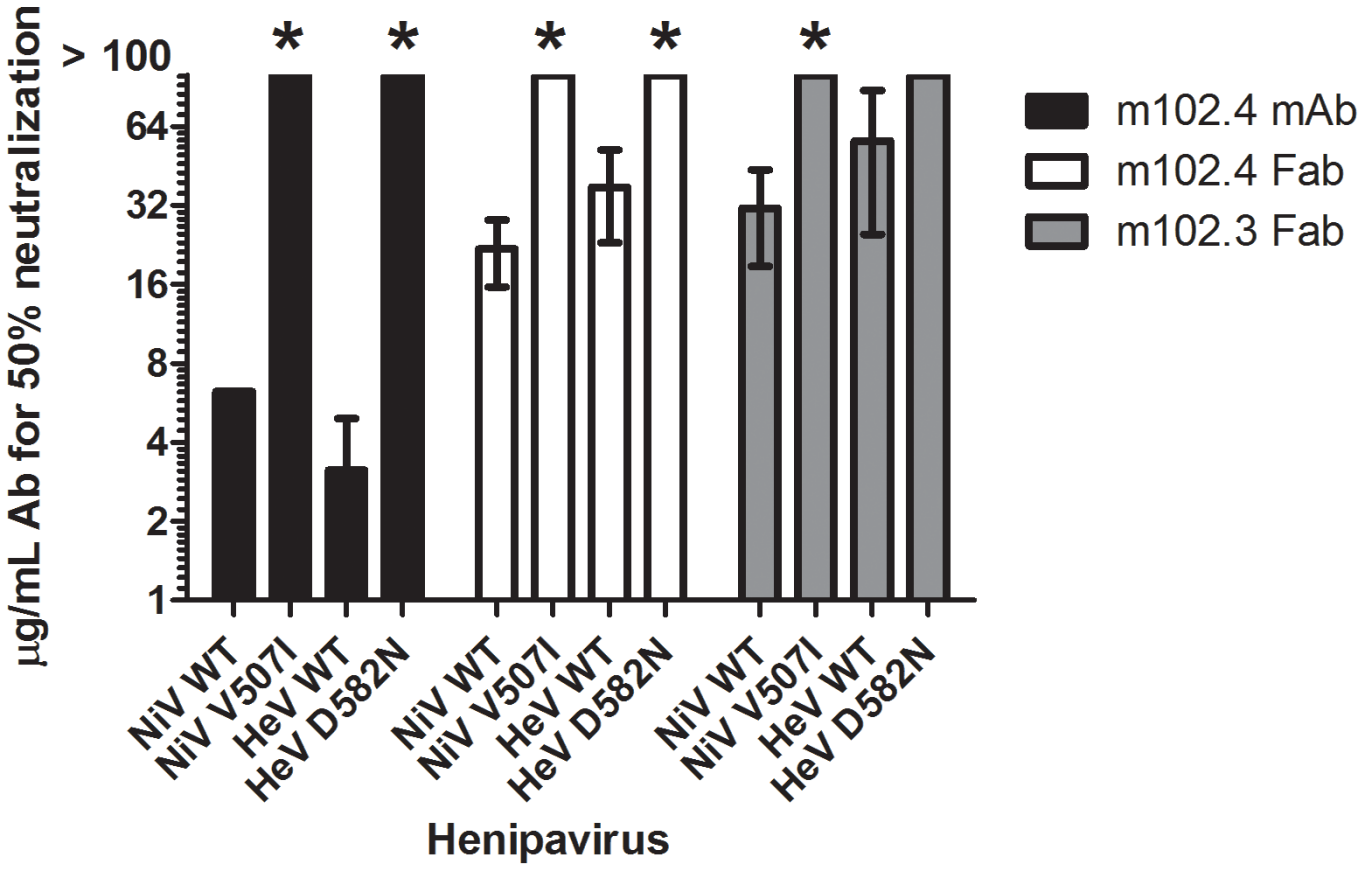
Neutralization efficacy of wild-type and neutralization-escape mutants by m102.4 mAb, m102.4 Fab and m102.3 Fab. Wild-type (WT) Nipah (NiV) and Hendra (HeV) viruses and their respective m102.4 neutralization escape mutants NiV-V507I and HeV-D582N were used to evaluate the neutralization efficacy of m102.4 mAb, m102.4 Fab and m102.3 Fab. Starting concentration of m102.4 was 100 µg/mL. The mAb and Fab concentrations at which 50% of the virus was neutralized are plotted. Error bars represent standard deviations. * t-test; p<0.001 compared to WT.

The panel of m102 derivatives was generated by light chain shuffling and heavy chain random mutagenesis. Among them, m102.4 was reported to have an equal or slightly higher affinity to henipavirus G glycoproteins in comparison to the others [19]. BioLayer Interferometry (Table S2), on the other hand indicates that m102.3 actually has slightly higher binding affinities to both NiV-G (K_D_: 5.6 nM) and HeV-G (K_D_: 27.4 nM) than m102.4 (K_D_: 25.5 nM and 111 nM, respectively). The primary sequences of m102.4 and m102.3 are overall highly similar, featuring an identical heavy chain (which provides all but one of the binding residues) and 15 different light chain amino acid that are not part of the m102.3/HeV-G interface (Figure S6) (but indirectly account for the small differences in binding affinities). As all HeV-G contacting residues in m102.3 are conserved in m102.4, the structural information obtained from the m102.3/HeV-G complex could also be applied to explain the mechanism of m102.4 neutralization. In the neutralization assay we performed, the efficiency of the m102.4 mAb to wild type NiV and HeV is three folds higher than that of the m102.3 Fab and m102.4 Fab, highlighting the importance of dimerization conferred by the Fc region. Notably, the efficiency of the m102.3 Fab is within the same range or slightly lower than that of the m102.4 Fab (Figure 4), suggesting that the neutralization efficiency may also be affected by the other factors, such as protein stability or flexibility.

### Generation of virus escape mutants

**Figure 5:**
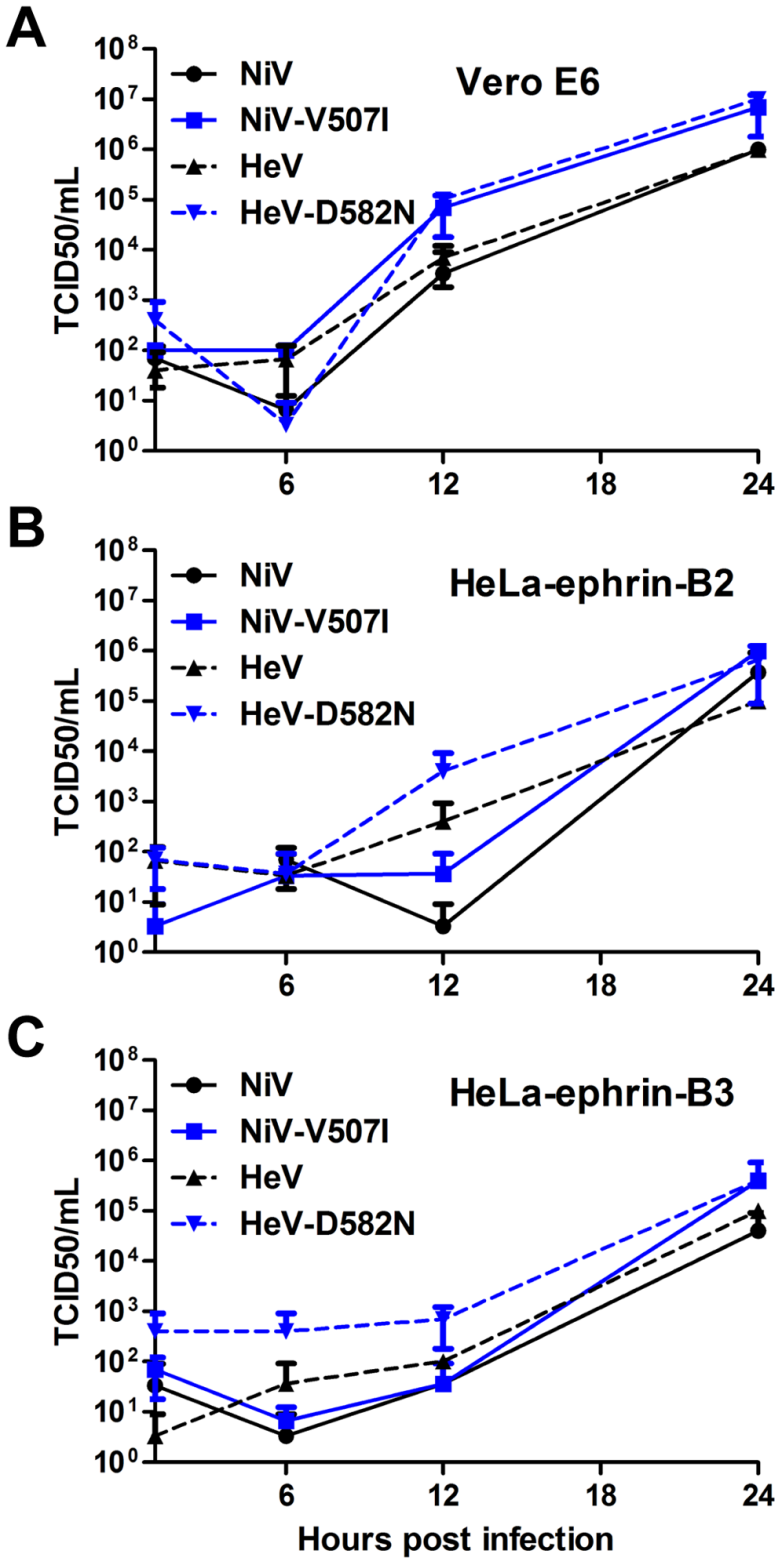
*In vitro* growth characteristics of wild type NiV and HeV and m102.4 neutralization escape mutants NiV-V507I and HeV-D582N. Cultures of (A) Vero E6, (B) HeLa-USU cells expressing human ephrin-B2 (HeLa-ephrin-B2) or (C) human ephrin-B3 (HeLa-eprhin-B3) were infected in triplicate with wild type and m102.4 neutralization escape mutants at a multiplicity of infection of 1 as described in Materials and Methods. Virus titers at different time points were determined by TCID_50_ using Vero E6 cells. Solid black line, NiV; solid grey line, NiV-V507I; dotted black line, HeV; dotted grey line, HeV-D582N. Error bars represent standard deviations.

To further detail and characterize the binding of the hmAb to the virus, infectious NiV and HeV were used to generate antibody neutralization escape mutants by incubating and culturing high titers of virus in the presence of hmAbs m102.3 (Fab fragment) or m102.4 (Fab fragment and mAb). After 3 passages, the resulting virus stocks were plaque purified and tested for neutralization efficacy. The G and F glycoprotein genes from a minimum of five plaques of each escape variant were sequenced in order to identify mutations associated with the antibody escape phenotype. For the NiV escape mutant, all ten plaques of both the m102.3 and m102.4 escape mutants contained a single amino acid change at location V507I. HeV mutants that escaped m102.4 neutralization all contained a single amino acid mutation at location D582N (Figure S5). The m102.3 and m102.4 cloned virus stocks of these escape mutants of NiV and HeV, in contrast to wild-type NiV and HeV, were no longer neutralized by m102.4 at antibody concentrations exceeding 100 µg/ml (Figure 4). In addition, the cloned virus stocks of the escape mutants were then analyzed in single round growth assays in comparison to wild-type HeV and NiV on Vero E6, HeLa-USU-ephrin-B2 and HeLa-USU-ephrin-B3 cells (Figure 5). Both neutralization escape mutants grew as efficiently and to equal titers as the wild-type virus in Vero E6 cells. Noticeably however, during passaging, the m102.3 and m102.4 neutralization resistant viruses were relatively slow in developing cytopathic effects (CPE). Whole genome sequence was performed to identify any additional mutations in these two escapes, as compared to their parent strains (sequencing report is attached in Supporting Materials as Report S1). The HeV escape variant contains another silent mutation in the P gene, in addition to the D582N mutation in the G gene; while the NiV escape variant contains mutations in the N gene (3′ end), M gene and L gene, in addition to the V507I mutation in the G gene. Thus, the escape is certainly due to the mutation in the G gene in case of the D582N HeV escape mutant.

Whole genome analysis of the parent virus stocks and the escape mutants and identification of all SNPs, indicates that the likely reason for the slow appearance of CPE in the presence of m102.4 was the need for resistant virus amplification to levels sufficient for cell-cell fusion.

Combined with binding affinity measurements (Table S2), the HeV-G/m102.3 structure provides clues to the escape mechanism of the escape mutants (Figure 2C and D). Interestingly, the affinity of the G proteins to both antibodies and ephrin-B2 was increased by the V507I mutation in NiV-G, and decreased by the D582N mutation in HeV-G. Residue V507I is located at the bottom of the NiV-G cavity, interacting with P119 of ephrin-B2, B3 and P107 of m102.3. The additional methyl group of I507 would likely result in a more intimate interaction with both the CDR-H3 and G-H loops resulting in a lower K_D_ value due to a decreased dissociation (k_off_) rate. Furthermore, ephrinB2 binding benefits slightly more than both antibodies from the V507I substitution. Intriguingly, D582 is located on the B6S2-S3 loop of the G protein, which is outside of the receptor/mAb binding region, suggesting the D582N mutation affects the Fab/G-protein interaction through an indirect pathway (Figure S5). D582 forms salt-bridges with two residues on B6S3, R589 and K591. The D582N mutation would likely cause a conformational change in B6S3, causing re-arrangement of several m102.3-contacting residues including I580, Y581 and I588, thus hindering the insertion with both the CDR-H3 and G-H loops. Indeed, the observed association rates (k_on_, Table S2) of this mutant to both antibodies and ephrinB2 decreased similarly. However, such a conformational change would affect the overall ephrin binding less, and it seems a similar rearrangement takes place even in the wild-type G protein (Figure S5) [9], [23]. Indeed, I580, Y581 and I588 are the pocket-forming residues for F117 of ephrinB2 (Figure 2D). The elimination of the two salt-bridges resulting from the D582N substitution might even have a slight stabilizing effect on the G-H loop insertion. Accordingly, the observed dissociation rate (k_off_) for binding of the HeV-G mutant (D582N) to ephrin-B2 was slightly decreased, while the dissociation rates for binding the antibodies were increased. In summary, both mutations in the G protein favor ephrin-B2 binding as compared to mAb binding, consistent with their neutralization-escape phenotypes.

## Discussion

Targeting the viral surface spike proteins has been a powerful strategy in the development of neutralizing mAbs. Similar to m102 targeting the henipavirus G proteins, a number of potent antibodies have been developed against the spike (S) glycoprotein of the SARS-associated coronavirus, the hemagglutinin glycoprotein of influenza virus and the envelope glycoprotein of HIV. The epitopes recognized by these antibodies are often functionally associated with the viral entry mechanism (e.g. attachment and membrane fusion) to reduce the occurrence of escape mutants. For instance in HIV, the epitopes targeted by neutralizing antibodies are located in four regions: receptor binding site (RBS), fusion associated membrane-proximal external region (MPER) region, conserved glycan structures and glycan associated loop regions, while in influenza, the epitopes are located in the fusion associated stem region and sialic acid binding pocket region [24]–[26]. In henipaviruses, as in all members of the paramyxovirus family, the attachment and fusion functions are exerted by two different proteins, which renders as possible epitope locations the RBS, the fusion-related regions, and sites associated with transducing the fusion-triggering signal from the attachment to the fusion proteins. In the past years, crystal structures of complexes between viral RBS and neutralizing antibodies have been determined, including m396 and 80R targeting the SARS S glycoprotein RBS [27], [28], CH65 and C05 targeting the influenza virus sialic acid binding pocket [24], [29], and b12, HJ16, VRC01, NIH45-46, 12A12, 3BNC117, VRC-PG04 and VRC-CH31 targeting the HIV-1 CD4-binding site (reviewed in [26]). Interestingly, in many of the examples above, the antibody CDR region mimics the conformation of the binding region of the cellular receptor (either protein or carbohydrate). Amongst them, most similar to our m102.3/HeV-G structure are the structures of CH65 and C05 targeting the influenza virus sialic acid binding site, which is a conserved shallow groove. All three mAbs use only CDRH3 to bind their target groove, but compared to CH65, C05 contacts a larger conserved region in the RBS, without interacting with the surrounding variable regions, which accounts for its greater neutralization breadth. The same strategy could be applied to further improve m102.3/4.

The m102.3/4 antibodies feature a long CDR-H3 (23 residues in kabat numbering), adapting a β-hairpin, providing an interesting example of how antibodies circumvent obstacles in reaching the targeted epitope. One of the challenges in viral epitope targeting is that the epitopes are sometimes hidden, either behind heavy glycosylation or deep in a cavity. Another example of a long CDR-H3 forming a β-hairpin is the antibody 2909 against HIV (21 residues) [30]. The extreme cases in this category are the HIV neutralizing antibodies PG9 and PG16, which contain a 28-residue axe-shaped CDR-H3 [31], [32].

Of the many tested therapeutic strategies to prevent and/or treat infection and disease caused by the henipaviruses in a variety of well-characterized animal models, few have been effective [33], [34]. Recently, the only post-exposure therapeutic option that is highly effective in animal models with clear potential for future approved human use applications has been the hmAb m102.4 [20], [21]. The reported success of m102.4 in a nonhuman primate model of HeV infection has been particularly encouraging, and the m102.4 exhibited an excellent distribution half-time (∼1 day) and elimination half-time (∼11 days) in the AGM. No evidence of HeV-specific pathology was observed in any of the m102.4-treated animals and no infectious HeV could be recovered. This study revealed that hmAb m102.4 prevented wide-spread HeV dissemination in virus challenged subjects, and was the first successful post-exposure *in vivo* therapy against HeV and the first in a nonhuman primate [21]. During the 2010 HeV spillover occurrence in Queensland, Australia, there were two individuals that were considered to be at high risk of HeV infection [35]. The m102.4 hmAb was requested by Australian health authorities and administered to the two individuals as a compassionate use therapeutic option even though no human safety testing has been carried out and it was not recommended for use in humans. In this instance, m102.4 was administered to the individuals prior to any HeV diagnosis or onset of clinical disease [35] with doses (∼19 mg/kg) sufficient to achieve a high serum concentration, and to date both individuals remain healthy and no evidence of HeV infection has been reported. The antibody appeared well tolerated when administered which was not unexpected since m102.4 is a human mAb. The m102.4 hmAb is now in further pre-clinical development stages in both the United States and Australia.

As part of our continued characterization of m102.4 we sought to provide the molecular details of its ephrin receptor blocking activity by determining the crystal structure of a (nearly identical) m102.4 derivative, m102.3, which possesses the same cross-reactive neutralizing and henipavirus G binding activity with an identical heavy chain sequence and G glycoprotein binding loop in the CDR3 domain. The structure reported here of the HeV-G/m102.3 complex reveals the molecular mechanism underling the exceptional cross-reactivity and neutralizing potency of these antibodies. The binding of the hmAb to the G glycoprotein involves a single loop of its heavy chain with hydrophobic amino acid residues occupying the same pockets in G that the ephrin receptors engage during receptor binding. It is clear now that the central cavity on the henipavirus G glycoprotein receptor-binding face is vital for viral attachment and infection. From the crystal structure of the m102.3/G protein complex, we know that blocking access to this cavity is a feasible and efficient way of inhibiting henipavirus attachment and infection. Specific peptide or small-molecule inhibitors for the viral attachment glycoprotein can be designed based on the structural data. For example, the existing pockets in the G glycoprotein cavity can be used as targets in structure-based computational screens. Another approach would be to screen compound libraries using a protein interaction primary assay, and then optimize the initial hits to better fit the binding cavity.

The data presented here are consistent with the initial steps of the henipavirus entry models proposed earlier based on the analysis of the G glycoprotein and the ephrin receptor/G glycoprotein complex structures [23], [36]–[38]. Of further importance, the new hmAb 102.3/G complex structure provides important information and leads for potential antibody improvement in two regards: increasing the antibody's affinity to the G glycoprotein in order to obtain even higher efficiency, and manipulating the interacting interface in order to reduce the potential of occurrence of escape mutants. The difficulty of the second aspect lies in the observation that the affinity of the attachment proteins to their receptors is not strictly correlated with the infection efficiency of henipaviruses. Thus, mutations that affect the henipavirus G glycoprotein binding affinities to ephrin receptors and mAbs to similar degrees could still allow potential escape. We indeed observed that even though there was a remarkable overlap between the m102.3 epitope and the receptor binding region of henipavirus G, two escape mutant variants of HeV and NiV, containing G glycoprotein mutations D582N and V507I respectively, were identified. *In vitro* manipulation, such as repeated passaging of virus and allowing replication in the presence of a neutralizing antibody is routinely used as an approach to generate escape variants that can then be examined as a means to map epitopes and detail mAb neutralization mechanisms. However, it should be emphasized that the appearance of m102.4 escape variants has not been observed in any of the *in vivo* efficacy testing against HeV or NiV to date, and this is likely explained by the fact that very high doses of mAb are utilized, similar to mAb dosing used in people in the prophylactic treatment of RSV infection with F (Synagis/Palivizumab) [39]. In addition, the effectiveness of m102.4 appears to be by virtue of its ability to slow the progression and dissemination of virus within the challenged host, allowing the host an effective window in which to mount its own innate and adaptive immune response that eventually prevents lethal disease outcome.

Taken together, the success of hmAb m102.4 *in vivo* as an effective post-exposure treatment against henipavirus disease in two different well-characterized animal models (the ferret and nonhuman primate), along with the new detailed structural findings on its viral G glycoprotein binding features that help explain its superior cross-reactive neutralizing activity, will facilitate efforts aimed at obtaining approved human use application to treat accidental exposure to HeV or NiV infection.

## Materials and Methods

### Protein expression and purification

Soluble head domain (amino acid residues 171-602) of HeV-G was cloned into a pGP67 vector and expressed in the Baculovirus expression system (BD Biosciences). The plasmid was transfected into SF9 cells using Cellfectin (Invitrogen) and Baculo-Gold linearized Baculovirus DNA (BD Biosciences). The virus was then amplified in SF9 cells for three rounds to reach the proper titer before applying to Hi5 cells for final expression (in 1∶100 volume infection ratio). The infected Hi5 cells were harvested 48 hours after infection. The cell media containing the HeV-G protein was purified using ion-exchange and size-exclusion chromatography (GE Biosciences). Soluble Fab was expressed and purified as described [18], [19]. The HeV-G/m102.3 complex was obtained by mixing the two proteins in a 1∶1.5 molar ratio and was passed through a Superdex 200 column (GE Biosciences). The fractions containing both proteins were collected and concentrated to 10 mg/ml in HBS buffer (20 mM Hepes pH 7.2, 10 mM KCl).

### Crystallization and structure determination

The initial crystallization condition was obtained with Wizard III (Emerald Biosystems) and Pro-complex (Qiagen) screens using robot screening (TTP LabTech's Mosquito). After several rounds of optimization using hanging drop vapor diffusion at room temperature, two crystals forms were obtained in conditions: 17% PEG 3350, 0.18M (NH_4_)_3_Citrate, and 15% PEG4000, 0.1M Hepes pH 7.0, 0.1M MgCl_2_. Crystals were frozen in liquid Nitrogen with 20% glycerol as cryo-protectant. Diffraction data were collected at beamline NE-CAT ID-24 of the Advanced Photon Source at Argonne National Laboratory. Data images were processed using program HKL2000. The structures were determined by molecular replacement with PDB ID 3D11 (NiV-G) and 1RZ1 (hmAb against GP120 of HIV virus). Phaser [40], COOT [41] and PHENIX REFINE in the program suite PHENIX [42] were used for structure determination, model building and refinement. The details of the crystallographic analysis are presented in Table S1.

### Escape mutant analysis

Neutralization resistant NiV and HeV mutants were generated by incubating 1×10_5_ TCID50 of each virus with 100 µg or 10 µg of mAbs m102.3 (NiV) and m102.4 (NiV or HeV) respectively, in 100 µl media for 1 h at 37°C and then inoculated onto 10_6_ Vero E6 cells in the presence of mAbs at the same concentration. The development of cytopathic effect (CPE) was monitored over 72 h and progeny viruses harvested. MAb treatment was repeated two additional times with CPE developing slowly with each passage. Passage 3 viruses were plaque purified in the presence of mAbs and neutralization resistant viruses were isolated. Experiments were performed in duplicate and the glycoprotein and fusion protein genes of individual plaques from each experiment were sequenced. The neutralization titers between wild type and the neutralization resistant virus were determined by micro neutralization assay. Briefly mAb m102.4 and Fabs m102.3 and m102.4 were serially diluted two-fold, and incubated with 100 TCID_50_ of the wild type (WT) and neutralization resistant NiV or HeV for 1 h at 37°C. Virus and antibodies were then added to a 96-well plate with 2×10^4^ Vero E6/well in 4 wells per antibody dilution. Wells were checked for CPE 3 days post infection and the 50% neutralization titer was determined as the mAb concentration at which at least 50% of wells showed no CPE.

### Virus growth curves

Growth curves were performed by inoculating cell cultures with NiV, HeV and their escape mutants at a multiplicity of infection (MOI) of 1 for 1 h, after which the cells were washed 3 times with PBS and overlaid with medium. Virus samples were obtained at various time points after infection and stored at −80°C until viral titers were determined by TCID_50_.

### Binding-affinity measurements

The binding kinetics of the wild type or mutant G proteins to both antibodies (m102.3 and m102.4) or to ephrin-B2 were measured by bio-layer interferometry on a BLItz instrument (ForteBio). Ni-NTA biosensors were used to immobilize the hexa-Histidine fused antibodies and ephrin-B2 proteins. Kinetic parameters (k_on_ and k_off_) and affinities (K_D_) were calculated from a non-linear fit of the BLItz instrument data using the BLItz software.

### Whole genome sequencing and analysis

250 µL of each virus was mixed with 750 µL Trizol LS and RNA was extracted following the manufacturers guidelines. Illumina TruSeq cDNA libraries were prepared from total RNA, omitting the polyA selection step. Each library was subjected to half a MiSeq run using a 300 cycle kit, paired end sequencing. A quality control tool for high throughput sequence, FASTQC, a java stand-alone program was downloaded from Babraham Bioinformatics Institute: http://www.bioinformatics.babraham.ac.uk/projects/fastqc/ and each fastq file was checked for quality. Resulting WT HeV and WT NiV reads were mapped to their respective reference genomes, NC_001906 and NC_002728, using CLC Genomics Workbench v6.0.4, using default parameters. Consensus sequence was extracted for each and used as the reference genome to which the reads resulting from sequencing the mutant samples were mapped. Consensus sequence for each mutant was extracted and aligned to the WT using CLC Genomics Workbench v6.0.4, and default parameters.

### Sequences alignment

Henipavirus G attachment glycoprotein sequences were aligned in CLC Main Workbench v5.7.1 using default parameters (gap open cost = 10; gap extension cost = 1).

### Illustrations

All molecular representations were produced with PyMOL (Delano Scientific LLC). Figures were prepared using Adobe Illustrator, Adobe Photoshop.

## Supporting Information

Figure S1 **Comparison of the Fab/HeV-G structures in the two crystal forms.** A: The m102.3/HeV-G complex structures in the two crystal forms were superimposed using the Fab as a reference. B: The complex structures were superimposed using HeV-G as a reference. Fab and HeV-G are colored in blue and grey in the P6_1_22 crystal form, and magenta and lime in the I222 crystal form, respectively.
(TIF)

Figure S2 **Electron density map at the m102.3 CDR-H3/HeV-G interface.** The interface between m102.3 CDR-H3 (magenta) and HeV-G (green) is illustrated. The contacting residues are labeled and shown in stick. The simulated annealing omit electron density map of the tip region of the m102.3 CDR-H3 is shown as blue mesh (contour level 3σ).
(TIF)

Figure S3 **Mapping previously identified HeV-G mutations that affect antibody binding.** The locations of all previously reported mutants are shown using the m102.3/HeV-G complex structure. Those affecting m102 binding are color in red, while those affecting m102.4 binding are colored in yellow. None of the residues are in direct contact with m102.3.
(TIF)

Figure S4 **Hetero-tetrameric packing of the m102.3/HeV-G complex.** The two HeV-G molecules (green) in the tetramer are connected by two Fab molecules. Fab1 (magenta heavy chain and cyan light chain) mainly binds to the HeV-G molecule that is on the bottom of the left panel and on the left side of the right panel. Fab2 (red heavy chain and blue light chain) mainly binds to the HeV-G molecule that is on the top of the left panel and on the right side of the right panel.
(TIF)

Figure S5 **Mechanism of the D582N m102.3/m102.4 escape mutant.** The m102.3/HeV-G complex viewed from the side around the B6 region of HeV-G. The HeV-G molecule is colored in green and the m102.3 molecule is colored in magenta. The HeV-G molecules in the ephrin-B2 bound state (grey) are superimposed with the m102.3 bound HeV-G molecule. D582 forms salt-bridges with R589 and K591 in both unbound and m102.3-bound HeV-G, but not when the molecule is bound to ephrin-B2. D582 of unbound and ephrin-B2-bound HeV-G is shown in thin stick. The B6S2-S3 loop of ephrin-B2-bound HeV-G sterically crashes with CDR-H3 of m102.3 upon superimposition of the HeV-Gs.
(TIF)

Figure S6 **Amino acid sequences alignment between m102.3 and m102.4.** The G-protein binding residues are highlighted in red. CDR-1, -2 and -3 of both the heavy and light chains are highlighted in blue.
(TIF)

Figure S7 **Amino acid sequences alignment between HeV-G and NiV-G.** The primary sequences of the HeV and NiV G proteins are aligned. The G glycoprotein residues interacting with mAb 102.3 (the epitope residues) are highlighted in red. These residues are conserved in all virus isolates reported in Genebank.
(TIF)

Report S1 **Henipavirus antibody escape sequencing report.**
(PDF)

Report S2 **Alignment of G proteins in all reported Hendra virus isolates in Genebank.**
(PDF)

Report S3 **Alignment of G proteins in all reported Nipah virus isolates in Genebank.**
(PDF)

Table S1 **Crystallographic data and refinement statistics.**
(DOC)

Table S2 **Affinity measurements of the mAb/G and ephrin-B2/G interactions performed using BioLayer Interferometry.** EFNb2 is ephrin-B2. A bar graph of the measured K_D_ values is also provided.
(DOC)
