# Supplementary figures and images for: Crystal Structure of the Hendra Virus Attachment G Glycoprotein Bound to a Potent Cross-Reactive Neutralizing Human Monoclonal Antibody
Source: PLoS Pathog. 2013 Oct 10;9(10):e1003684. doi: 10.1371/journal.ppat.1003684 (PMC3795035; doi:10.1371/journal.ppat.1003684)

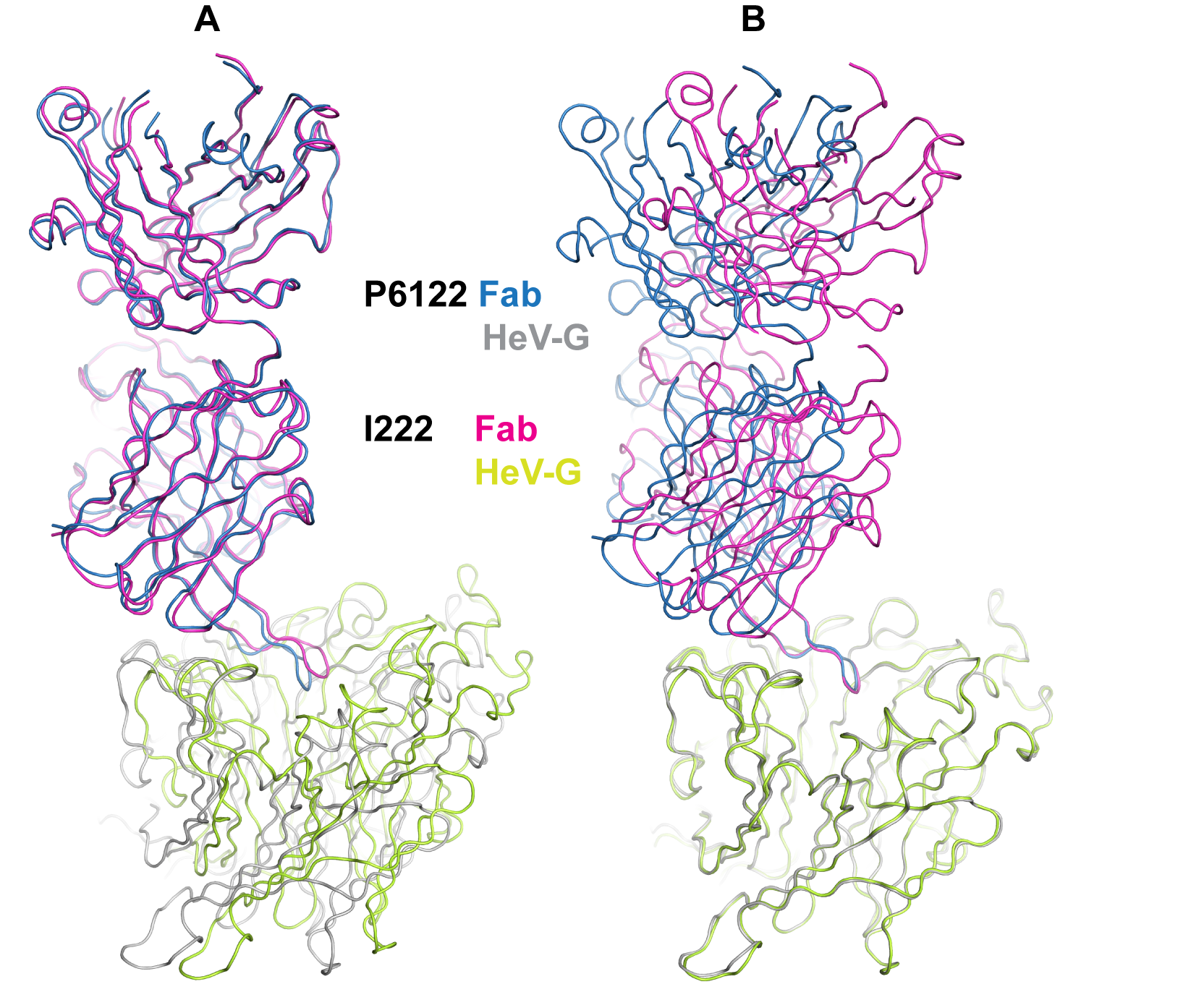

Supplement: Figure S1 — Comparison of the Fab/HeV-G structures in the two crystal forms. A: The m102.3/HeV-G complex structures in the two crystal forms were superimposed using the Fab as a reference. B: The complex structures were superimposed using HeV-G as a reference. Fab and HeV-G are colored in blue and grey in the P6122 crystal form, and magenta and lime in the I222 crystal form, respectively. (TIF) [file ppat.1003684.s001.tif]

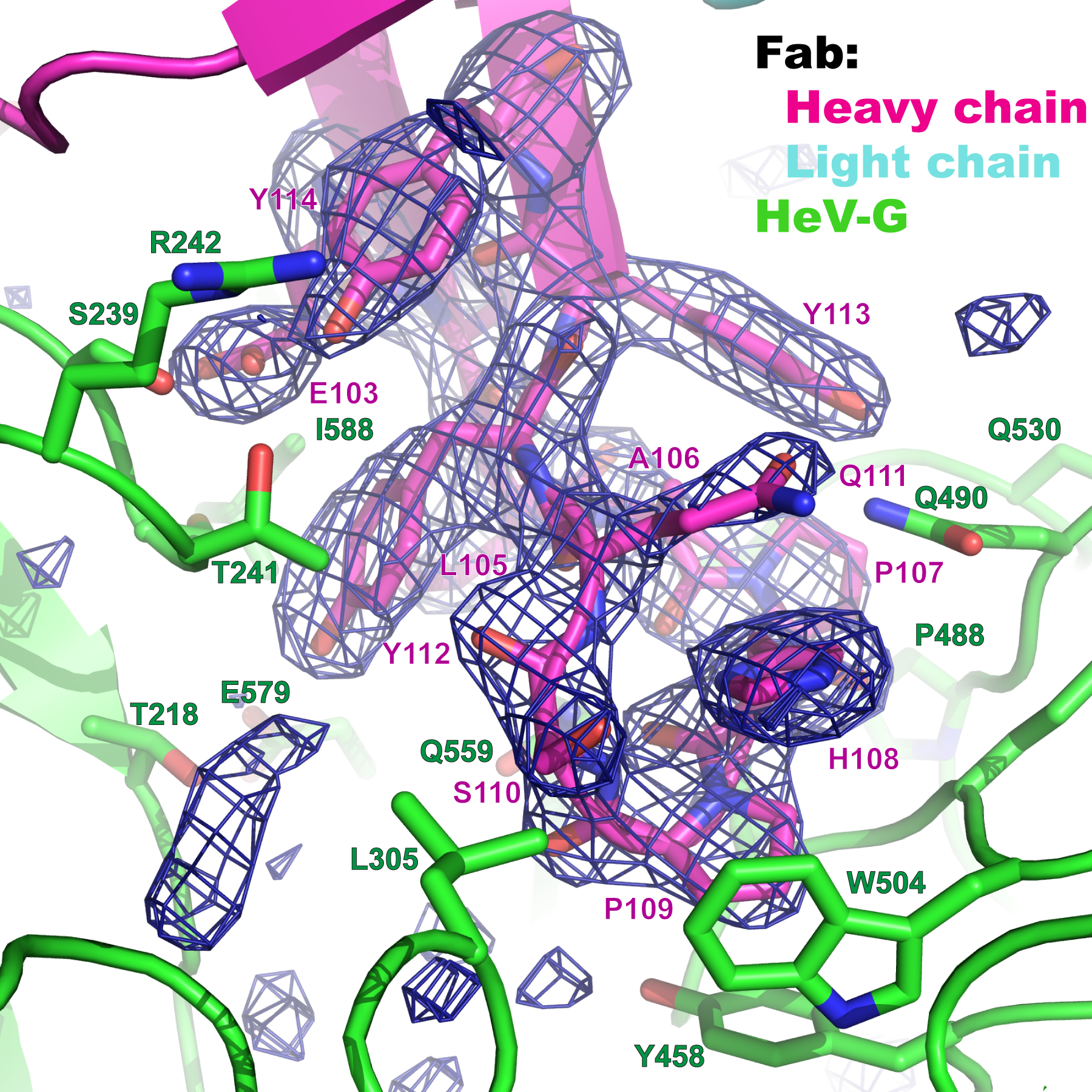

Supplement: Figure S2 — Electron density map at the m102.3 CDR-H3/HeV-G interface. The interface between m102.3 CDR-H3 (magenta) and HeV-G (green) is illustrated. The contacting residues are labeled and shown in stick. The simulated annealing omit electron density map of the tip region of the m102.3 CDR-H3 is shown as blue mesh (contour level 3σ). (TIF) [file ppat.1003684.s002.tif]

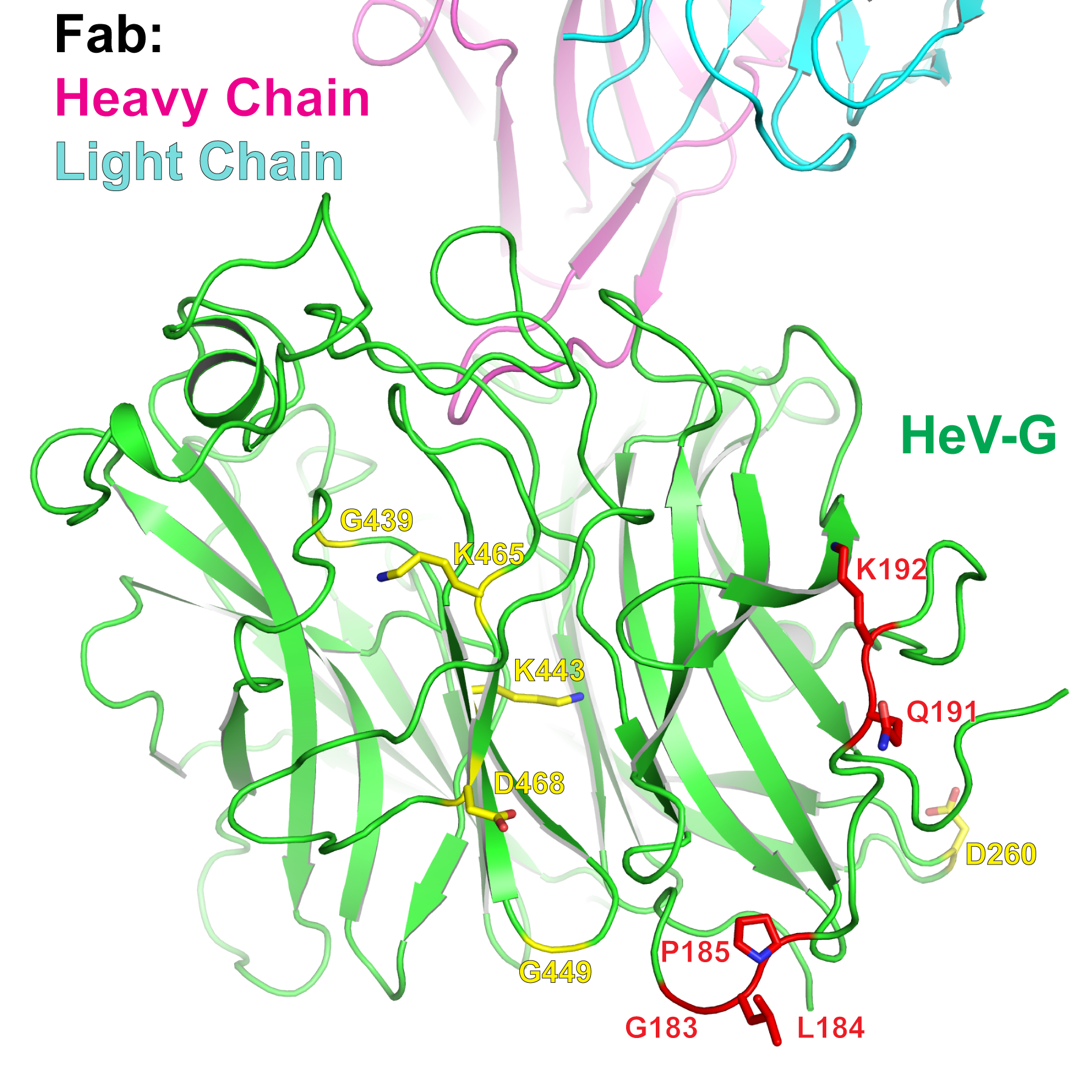

Supplement: Figure S3 — Mapping previously identified HeV-G mutations that affect antibody binding. The locations of all previously reported mutants are shown using the m102.3/HeV-G complex structure. Those affecting m102 binding are color in red, while those affecting m102.4 binding are colored in yellow. None of the residues are in direct contact with m102.3. (TIF) [file ppat.1003684.s003.tif]

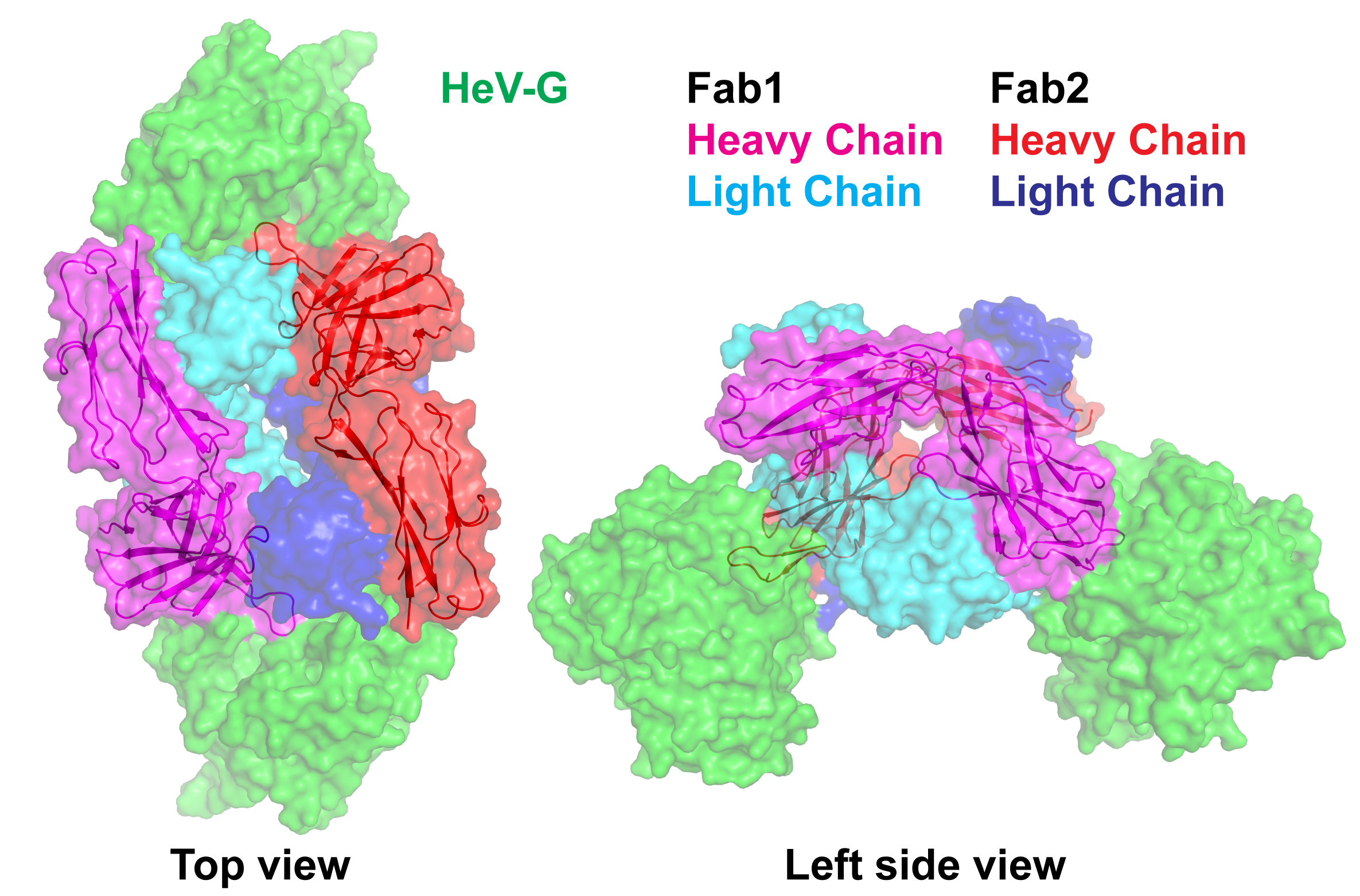

Supplement: Figure S4 — Hetero-tetrameric packing of the m102.3/HeV-G complex. The two HeV-G molecules (green) in the tetramer are connected by two Fab molecules. Fab1 (magenta heavy chain and cyan light chain) mainly binds to the HeV-G molecule that is on the bottom of the left panel and on the left side of the right panel. Fab2 (red heavy chain and blue light chain) mainly binds to the HeV-G molecule that is on the top of the left panel and on the right side of the right panel. (TIF) [file ppat.1003684.s004.tif]

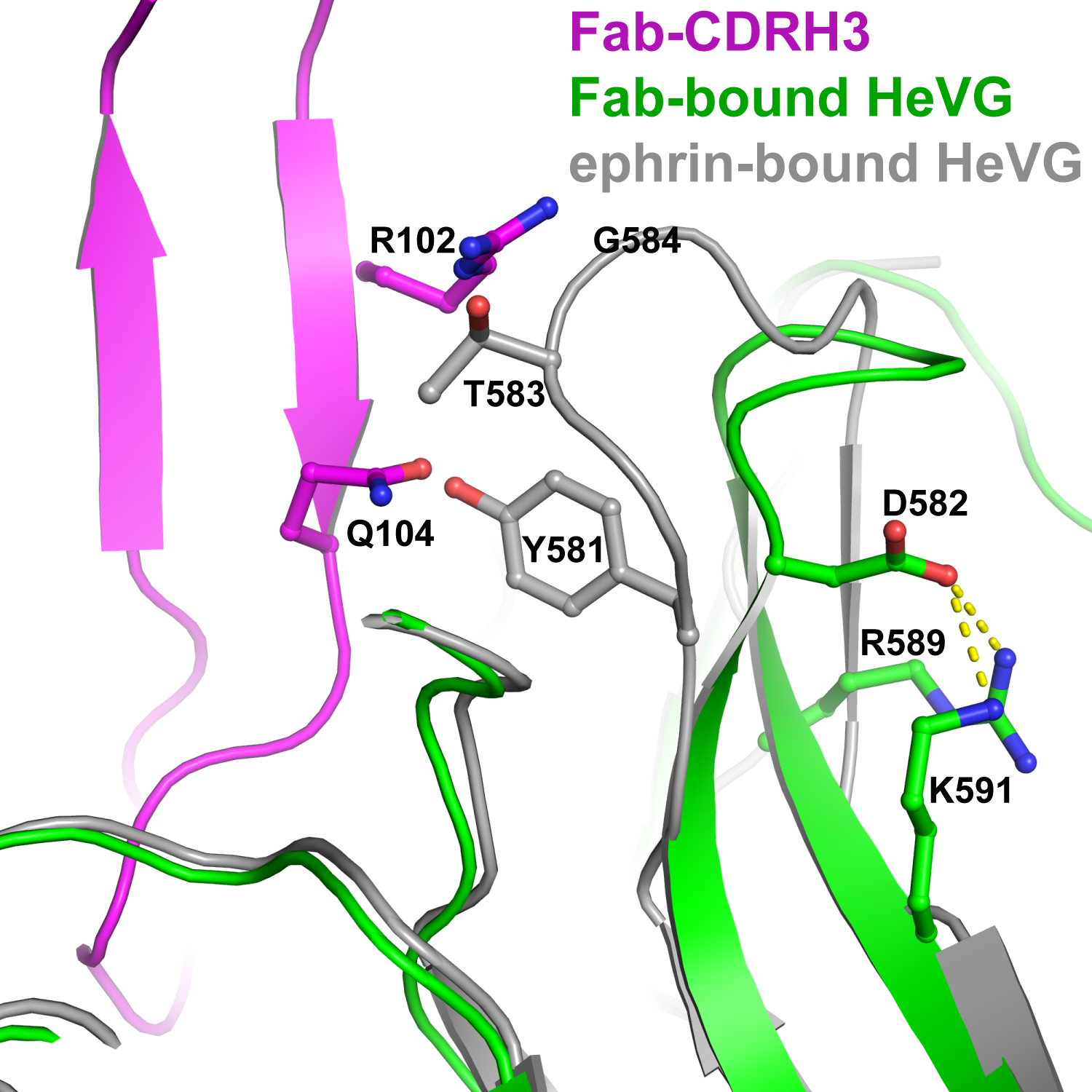

Supplement: Figure S5 — Mechanism of the D582N m102.3/m102.4 escape mutant. The m102.3/HeV-G complex viewed from the side around the B6 region of HeV-G. The HeV-G molecule is colored in green and the m102.3 molecule is colored in magenta. The HeV-G molecules in the ephrin-B2 bound state (grey) are superimposed with the m102.3 bound HeV-G molecule. D582 forms salt-bridges with R589 and K591 in both unbound and m102.3-bound HeV-G, but not when the molecule is bound to ephrin-B2. D582 of unbound and ephrin-B2-bound HeV-G is shown in thin stick. The B6S2-S3 loop of ephrin-B2-bound HeV-G sterically crashes with CDR-H3 of m102.3 upon superimposition of the HeV-Gs. (TIF) [file ppat.1003684.s005.tif]

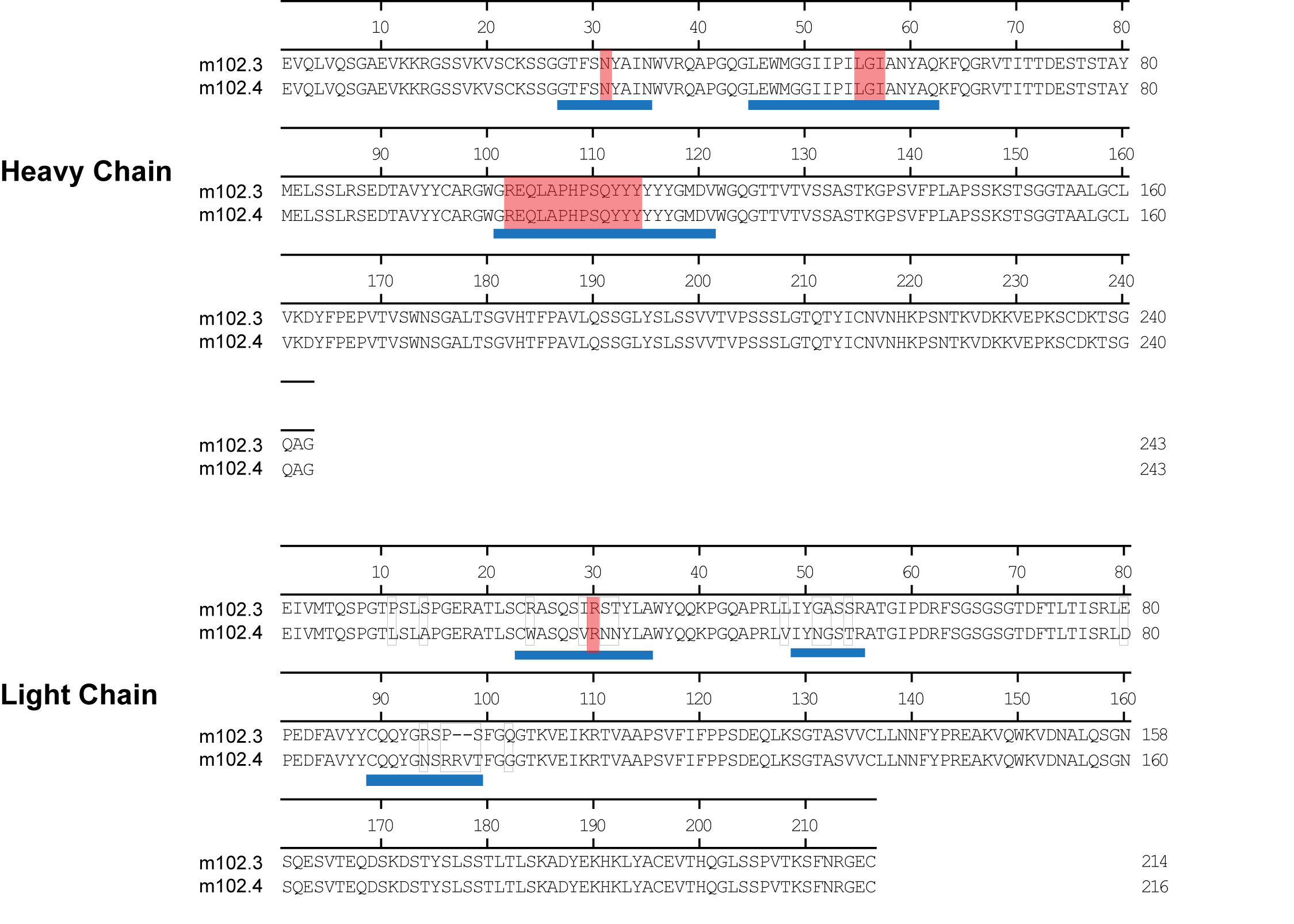

Supplement: Figure S6 — Amino acid sequences alignment between m102.3 and m102.4. The G-protein binding residues are highlighted in red. CDR-1, -2 and -3 of both the heavy and light chains are highlighted in blue. (TIF) [file ppat.1003684.s006.tif]

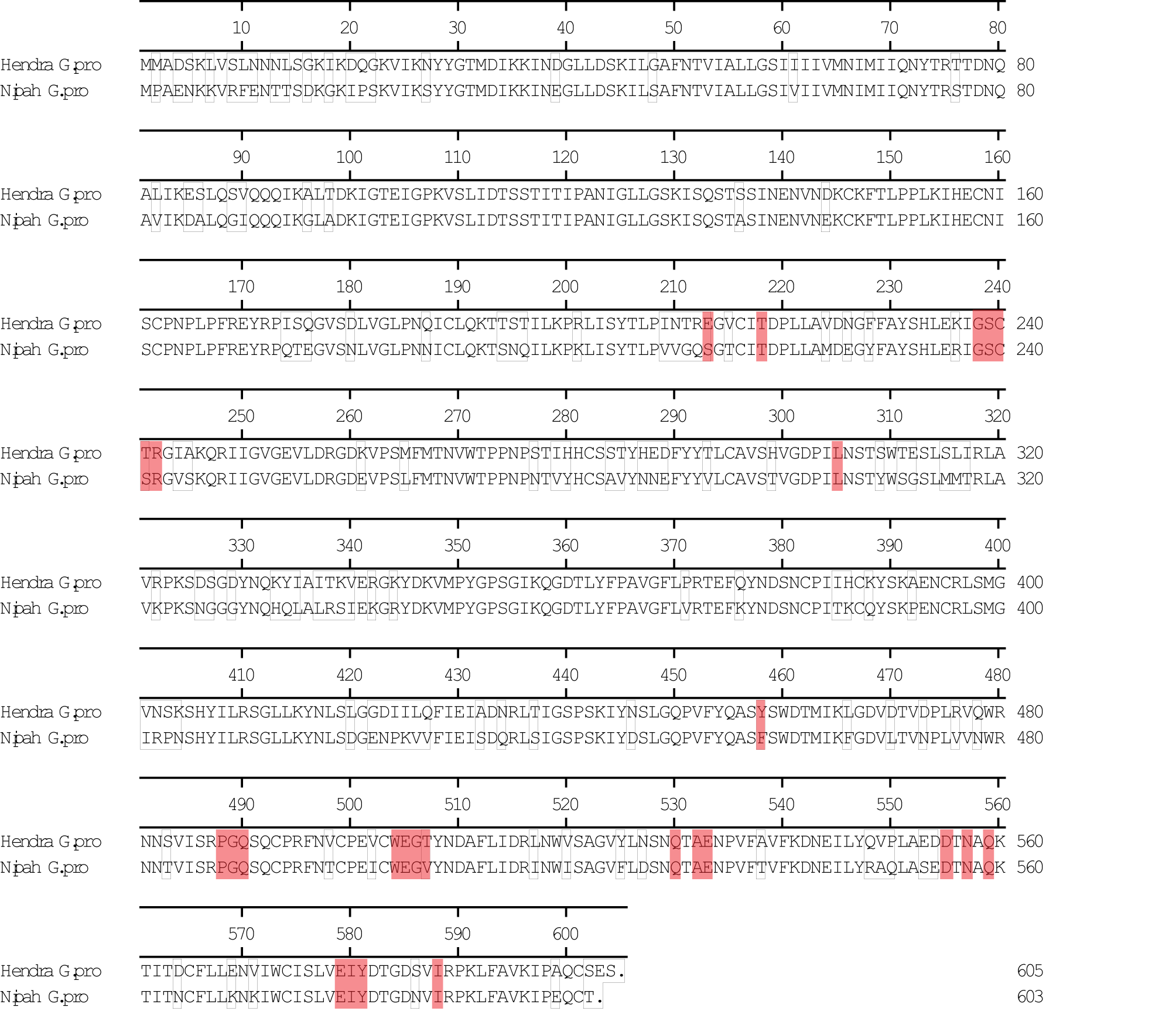

Supplement: Figure S7 — Amino acid sequences alignment between HeV-G and NiV-G. The primary sequences of the HeV and NiV G proteins are aligned. The G glycoprotein residues interacting with mAb 102.3 (the epitope residues) are highlighted in red. These residues are conserved in all virus isolates reported in Genebank. (TIF) [file ppat.1003684.s007.tif]
