# Supplementary material for: Crystal Structure of the Hendra Virus Attachment G Glycoprotein Bound to a Potent Cross-Reactive Neutralizing Human Monoclonal Antibody
Source: PLoS Pathog. 2013 Oct 10;9(10):e1003684. doi: 10.1371/journal.ppat.1003684 (PMC3795035; doi:10.1371/journal.ppat.1003684)
Supplement: Report S1 — Henipavirus antibody escape sequencing report. (PDF) [file ppat.1003684.s008.pdf]

HENIPAVIRUS ANTIBODY ESCAPE SEQUENCING REPORT

Kimberly Bishop-Lilly[1,2], Truong Luu[1,2], Regina Cer[1,2], and LT Vishwesh Mokashi[1]
[1]Naval Medical Research Center, NMRC-Frederick, 8400 Research Plaza, Fort Detrick, MD 21702
[2]Henry M. Jackson Foundation, 6720-A Rockledge Drive, Suite 100, Bethesda, MD 20817

1. OBJECTIVE

To provide whole genome sequence (WGS) data for antibody escape mutants of Hendra virus (HeV) and Nipah virus (NiV) as well as the parent strains.

2. METHODS

2.1 Sequencing. Illumina TruSeq cDNA libraries were prepared from total RNA provided. Each library was subjected to half a MiSeq run using 300 cycle kit, paired end sequencing.

2.2 Quality control. A quality control tool for high throughput sequence, FASTQC, a java stand-alone program was downloaded from Babraham Bioinformatics Institute: http://www.bioinformatics.babraham.ac.uk/projects/fastqc/ and each fastq sequence was checked for quality.

2.3 Sequence analysis. Resulting WT HeV and WT NiV reads were mapped to their respective reference genomes, NC_001906 and NC_002728, using CLC Genomics Workbench v6.0.4, using default parameters. Consensus sequence was extracted for each and used as the reference genome to which the reads resulting from sequencing the mutant samples were mapped. Consensus sequence for each mutant was extracted and aligned to the WT using CLC Genomics Workbench v6.0.4, and default parameters.

3. RESULTS

3.1 Quality Control of FASTQ files. The FASTQ files passed quality control according to reports from FastQC software analysis.

3.1.1 Per Base Sequence Quality

This view shows an overview of the range of quality values across all bases at each position in the FastQ file. For each position a BoxWhisker type plot is drawn. The elements of the plot are as follows:

- The central red line is the median value
- The yellow box represents the inter-quartile range (25-75%)
- The upper and lower whiskers represent the 10% and 90% points
- The blue line represents the mean quality

The y-axis on the graph shows the quality scores. The higher the score the better the base call. The background of the graph divides the y axis into very good quality calls (green), calls of reasonable quality (orange), and calls of poor quality (red).

The quality of calls on most platforms will degrade as the run progresses, so it is common to see base calls falling into the orange area towards the end of a read. It should be mentioned that there are number of different ways to encode a quality score in a FastQ file. FastQC attempts to automatically determine which encoding method was used, but in some very limited datasets it is possible that it will guess this incorrectly (ironically only when your data is universally very good!). The title of the graph will describe the encoding FastQC thinks your file used.

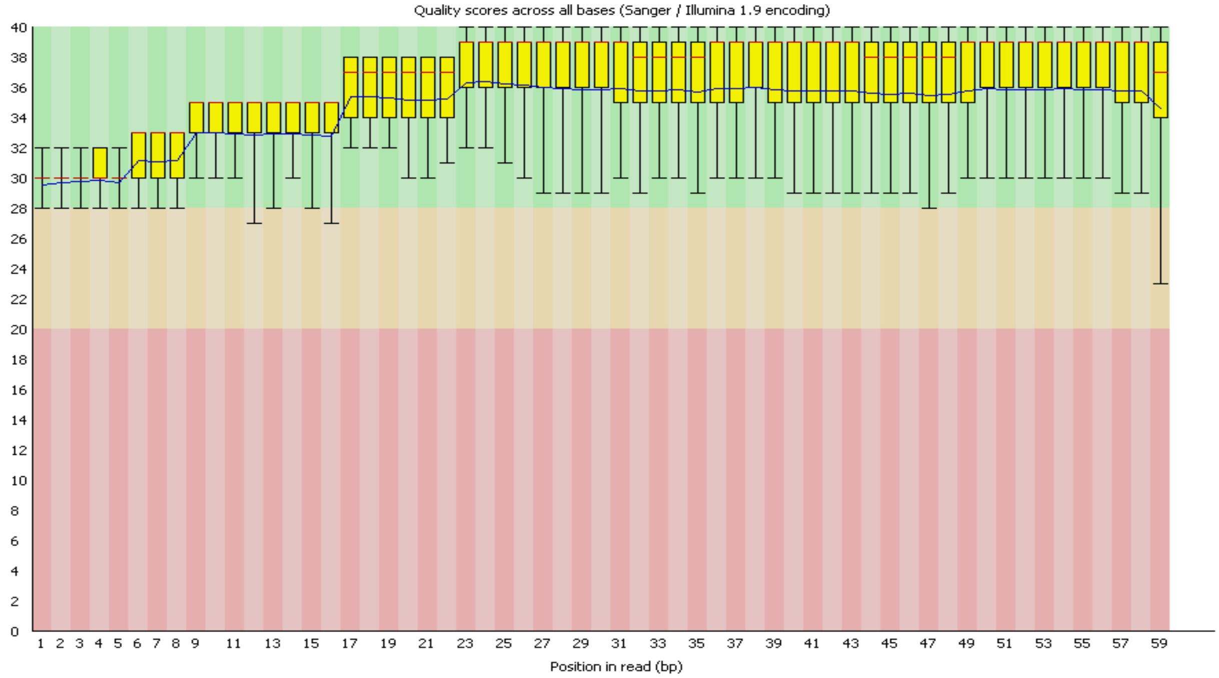

3.1.2 Per Sequence Quality Scores

The per sequence quality score report allows you to see if a subset of your sequences have universally low quality values. It is often the case that a subset of sequences will have universally poor quality, often because they are poorly imaged (on the edge of the field of view etc), however these should represent only a small percentage of the total sequences. If a significant proportion of the sequences in a run have overall low quality then this could indicate some kind of systematic problem - possibly with just part of the run (for example one end of a flowcell). A warning is raised if the most frequently observed mean quality is below 27 - this equates to a 0.2% error rate. An error is raised if the most frequently observed mean quality is below 20 - this equates to a 1% error rate.

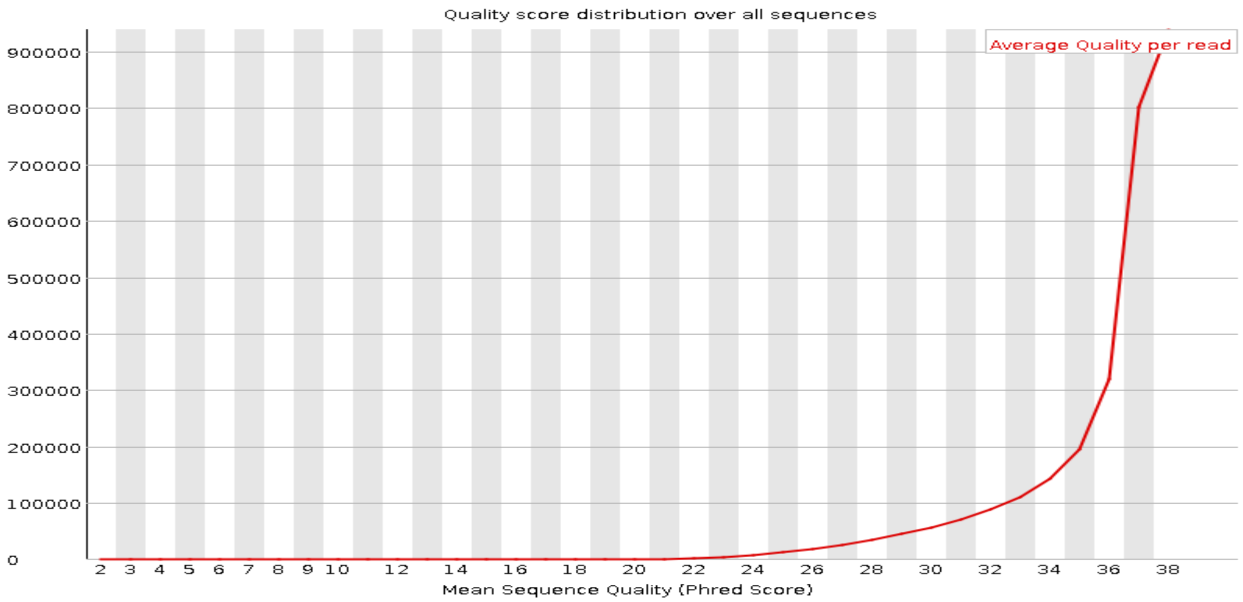

3.1.3 Per Base N Content

If a sequencer is unable to make a base call with sufficient confidence then it will normally substitute an N rather than a conventional base call. This module plots out the percentage of base calls at each position for which an N was called. It's not unusual to see a very low proportion of Ns appearing in a sequence, especially nearer the end of a sequence. However, if this proportion rises above a few percent it suggests that the analysis pipeline was unable to interpret the data well enough to make valid base calls.

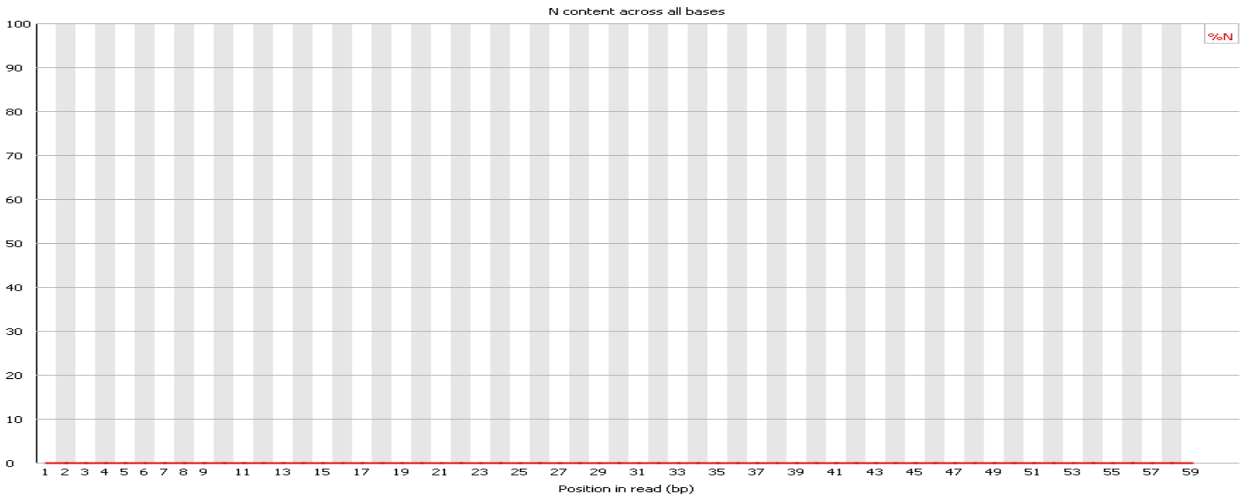

3.1.4 Per Sequence GC Content

This module measures the GC content across the whole length of each sequence in a file and compares it to a modelled normal distribution of GC content.

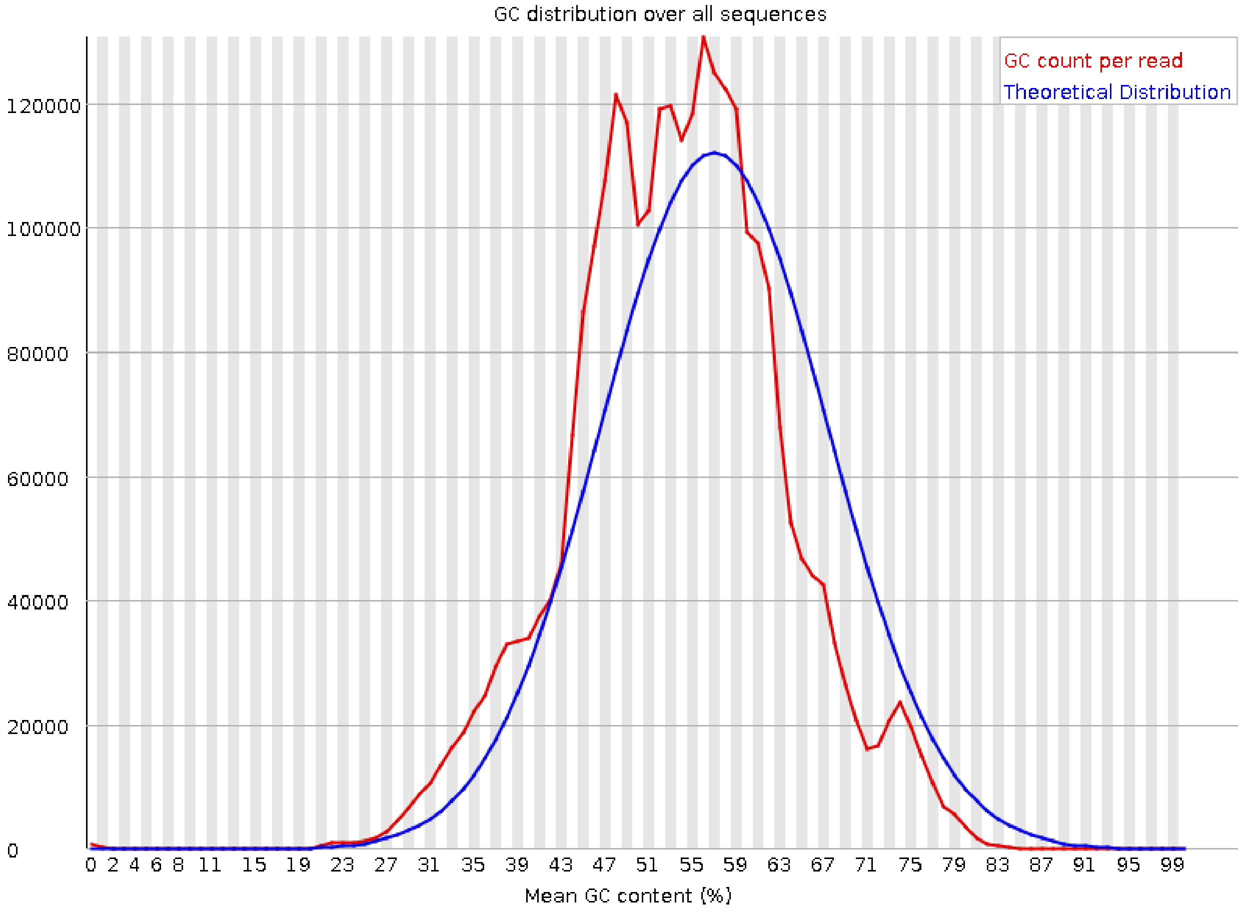

In a normal random library you would expect to see a roughly normal distribution of GC content where the central peak corresponds to the overall GC content of the underlying genome. Since we don't know the the GC content of the genome the modal GC content is calculated from the observed data and used to build a reference distribution.

An unusually shaped distribution could indicate a contaminated library or some other kinds of biased subset. A normal distribution which is shifted indicates some systematic bias which is independent of base position. If there is a systematic bias which creates a shifted normal distribution then this won't be flagged as an error by the module since it doesn't know what your genome's GC content should be. A warning is raised if the sum of the deviations from the normal distribution represents more than 15% of the reads. This module will indicate a failure if the sum of the deviations from the normal distribution represents more than 30% of the reads.

3.2 Sequence analysis.

3.2.1 Hendra virus

The quality-controlled, paired reads resulting from sequencing the WT HeV sample were mapped to the publically available HeV reference genome, NC_001906. 2,631,464 of the total 14,340,856 reads mapped (16.47%). The unmapped reads presumably derive from the host cells the virus was grown in (and preliminary results indicate that the unaligned reads from these samples do indeed map to human or macaque genomes). 100% of the reference genome was covered by reads. Minimum depth of coverage at a given position was 4X and maximum depth of coverage was 36,534X. Average depth of coverage was 18,340.91X, with standard deviation of 4,880.24.

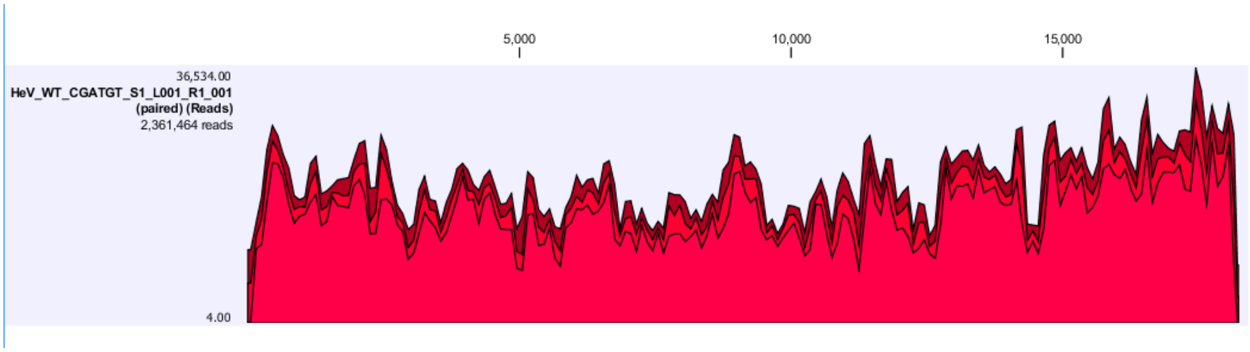

Above: Coverage of HeV genome by sequencing reads (WT).

When the resulting consensus sequence was compared to the reference, there were 12 nucleotide differences noted, which are described below.

Below: Differences in HeV consensus as compared to HeV reference NC_001906

Position (coordinate of ref)	reference	consensus
5,884	T	A
7,404	G	A
11,115	A	G
12,591	G	A
12,633	C	G
13,425	C	A
14,023	T	A
14,371	G	T
15,293˅15,294	-	C
15,376	G	-
15,474	G	T
15,475	C	G

The HeV WT consensus was then used as the reference genome to which the antibody escape mutant of HeV reads were mapped. 841,237 of 5,772,070 total reads mapped (14.57%). 100% of the reference genome was covered by reads. Minimum depth of coverage was 1X, maximum depth of coverage was 15,946X, and average depth of coverage was 6,552X, with standard deviation of 2,076.97.

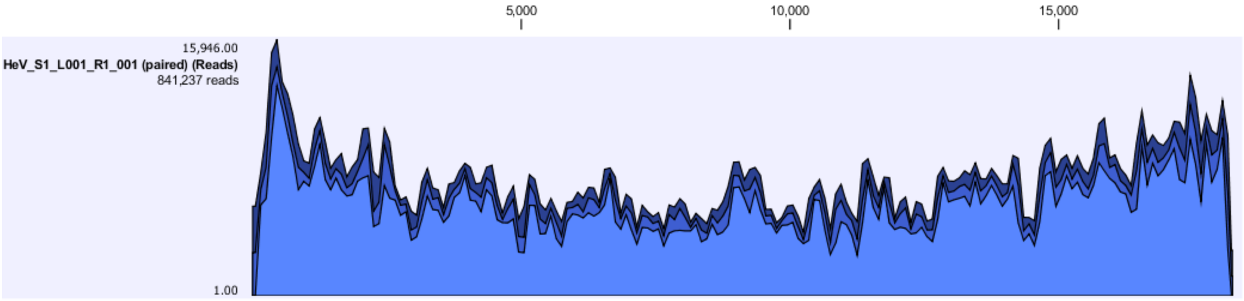

Above: Coverage of HeV genome by sequencing reads (mutant).

Two single nucleotide polymorphisms (SNPs) were identified in the HeV mutant as compared to the parent HeV, and they are described in the table below. The first SNP, in the P phosphoprotein gene, was found to be synonomous, while the second SNP was found to result in the mutation of residue 582 in the G glycoprotein from Aspartic acid to Asparagine.

Below: Differences in HeV mutant as compared to HeV WT

Position	WT	mutant	consequence
3,689	C	T	Silent, phosphoprotein P gene
10,656	G	A	D582N, glycoprotein G gene

3.2.2 Nipah virus. The quality-controlled, paired reads resulting from sequencing the WT NiV sample were mapped to the publically available NiV reference genome, NC_002728. 1,296,181 of the total 12,760,752 reads mapped (10.16%). As with the HeV sample, the unmapped reads presumably derive from the host cells the virus was grown in. 100% of the reference genome was covered by reads. Minimum depth of coverage at a given position was 7X and maximum depth of coverage was 17,440X. Average depth of coverage was 9,959.27X, with standard deviation of 2,238.30. When the resulting consensus sequence was compared to the reference, there were no differences noted.

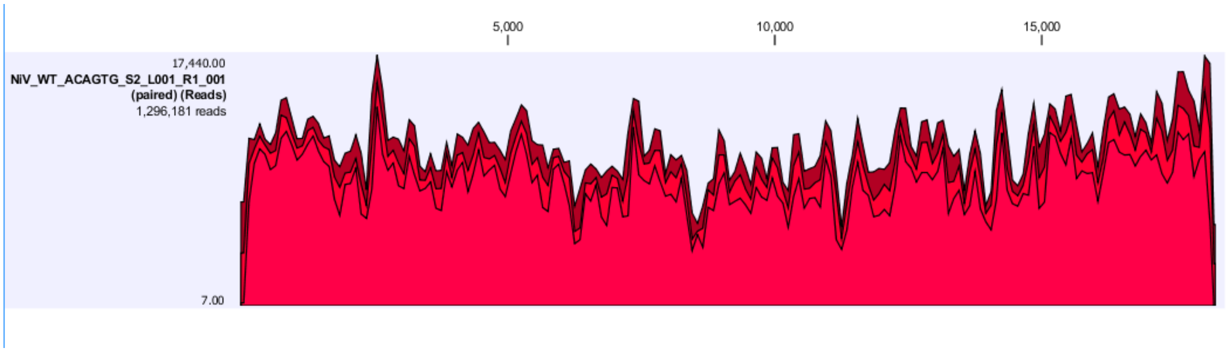

Above: Coverage of NiV genome by sequencing reads (WT).

The NiV WT consensus was then used as the reference genome to which the antibody escape mutant of NiV reads were mapped. 1,426,343 of 34,470,164 total reads mapped (4.14%). 100% of the reference genome was covered by reads. Minimum depth of coverage was 9X, maximum depth of coverage was 38,800X, and average depth of coverage was 10,654.07X, with standard deviation of 4,614.04.

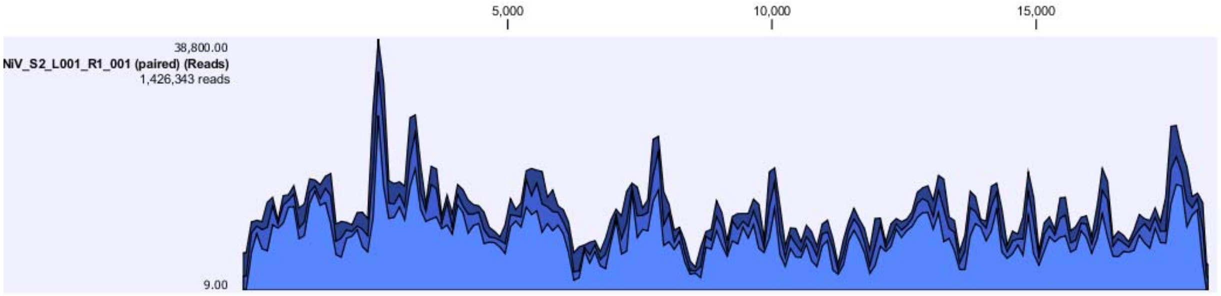

Above: Coverage of NiV genome by sequencing reads (mutant).

Seven SNPs were identified in the NiV mutant as compared to the parent NiV isolate, and they are described in the table below. Of the 7 SNPs identified in the mutant NiV genome, 3 were silent mutations in the polymerase gene, one of as yet unknown consequence was identified in the 3' region of the N gene in a region that doesn't get translated, and 3 mutations were identified that resulted in amino acid changes within translated regions of genes. Of these latter 3 mutations, one resulted in mutation of residue 192 of the matrix protein from Threonine to Serine, one resulted in mutation of residue 507 of the attachment glycoprotein G from Valine to Isoleucine, and one resulted in mutation of residue 1,946 of the polymerase from Aspartic acid to Asparagine.

Below: Differences in NiV mutant as compared to NiV WT

Position	WT	mutant	consequence
1,832	T	C	Unknown; outside translated region of N gene (3' end)
5,681	A	T	T192S, M gene
10,461	G	A	V507I, glycoprotein G gene
12,212	C	T	Silent, polymerase gene
12,300	C	T	Silent, polymerase gene
16,550	A	G	Silent, polymerase gene
17,247	G	A	D1,946N, polymerase gene

4. CONCLUSIONS

The D582N and V507I mutations in the Hendra and Nipah mutants' G glycoproteins respectively, are likely the mutations responsible for antibody escape because 1) the Heniparivus attachment

glycoprotein, G, is known to be the target for m102 binding and 2) the only other mutation found in the HeV mutant is a silent mutation in the phosphoprotein G and therefore unlikely to affect m102 binding.

Then HeV WT sample was found to have 12 differences as compared to the available reference in GenBank, NC_001906. These differences consist of 10 SNPs and 2 indels (insertions/deletions). As expected, all 12 of these differences were also found in the NiV antibody escape mutant which derived from that sample. Conversely, the NiV WT sample was found to be 100% identical to the available reference genome in GenBank, NC_002728. The NiV mutant was found to contain 6 SNPs in addition to the V507I mutation in the attachment glycoprotein G, and 2 of these additional SNPs result in amino acid changes which are not expected to affect m102 binding, but their effects on the viral life cycle, if any, are as yet unknown.

BISHOP-LILLY.KIMBERLY.ANN.1293249314

Digitally signed by BISHOP-LILLY.KIMBERLY.ANN.1293249314
DN: c=US, o=U.S. Government, ou=DoD, ou=PKI, ou=CONTRACTOR, cn=BISHOP-LILLY.KIMBERLY.ANN.1293249314
Date: 2013.06.21 10:32:28 -04'00'
