# Supplementary material for: Crystal Structure of the Hendra Virus Attachment G Glycoprotein Bound to a Potent Cross-Reactive Neutralizing Human Monoclonal Antibody
Source: PLoS Pathog. 2013 Oct 10;9(10):e1003684. doi: 10.1371/journal.ppat.1003684 (PMC3795035; doi:10.1371/journal.ppat.1003684)

```
                                                            20                        40
                                                             |                         |
gi|29432477|gb|AAC83193.2|   MMADSKLVSL NNNLSGKIKD QGKVIKNYYG TMDIKKINDG LLDSKILGAF  50
gi|328495173|gb|AEB21198.1|  MMADSKLVSL NNNLSGKIKD QGKVIKNYYG TMDIKKINDG LLDSKILGAF  50
gi|328495213|gb|AEB21234.1|  MMADSKLVSL NNNLSGKIKD QGKVIKNYYG TMDIKKINDG LLDSKILGAF  50
gi|350998758|gb|AEQ38062.1|  MMADSKLVSL NNNLSGKIKD QGKVIKNYYG TMDIKKINDG LLDSKILGAF  50
gi|350998768|gb|AEQ38071.1|  MMADSKLVSL NNNLSGKIKD QGKVIKNYYG TMDIKKINDG LLDSKILGAF  50
gi|350998778|gb|AEQ38080.1|  MMADSKLVSL NNNLSGKIKD QGKVIKNYYG TMDIKKINDG LLDSKILGAF  50
gi|350998842|gb|AEQ38133.1|  MMADSKLVSL NNNLSGKIKD QGKVIKNYYG TMDIKKINDG LLDSKILGAF  50
gi|350998851|gb|AEQ38141.1|  MMADSKLVSL NNNLSGKIKD QGKVIKNYYG TMDIKKINDG LLDSKILGAF  50
gi|29468607|ref|NP_047112.2| MMADSKLVSL NNNLSGKIKD QGKVIKNYYG TMDIKKINDG LLDSKILGAF  50
gi|328495182|gb|AEB21206.1|  MMADSKLVSL NNNLSGKIKD QGKVIKNYYG TMDIKKINDG LLDSKILGAF  50
gi|328495203|gb|AEB21225.1|  MMADSKLVSL NNNLSGKIKD QGKVIKNYYG TMDIKKINDG LLDSKILGAF  50
gi|350998831|gb|AEQ38124.1|  MMADSKLVSL NNNLSGKIKD QGKVIKNYYG TMDIKKINDG LLDSKILGAF  50
gi|350998718|gb|AEQ38026.1|  MMADSKLVSL NNNLSGKIKD QGKVIKNYYG TMDIKKINDG LLDSKILGAF  50
gi|350998728|gb|AEQ38035.1|  MMADSKLVSL NNNLSGKIKD QGKVIKNYYG TMDIKKINDG LLDSKILGAF  50
gi|350998737|gb|AEQ38043.1|  MMADSKLVSL NNNLSGKIKD QGKVIKNYYG TMDIKKINDG LLDSKILGAF  50
gi|350998747|gb|AEQ38052.1|  MMADSKLVSL NNNLSGKIKD QGKVIKNYYG TMDIKKINDG LLDSKILGAF  50
gi|350998803|gb|AEQ38099.1|  MMADSKLVSL NNNLSGKIKD QGKVIKNYYG TMDIKKINDG LLDSKILGAF  50
gi|350998821|gb|AEQ38115.1|  MMADSKLVSL NNNLSGKIKD QGKVIKNYYG TMDIKKINDG LLDSKILGAF  50
gi|350998813|gb|AEQ38108.1|  MMADSKLVSL NNNLSGKIKD QGKVIKNYYG TMDIKKINDG LLDSKILGAF  50
gi|328495193|gb|AEB21216.1|  MMADSKLVSP NNNLSGKIKD QGKVIKNYYG TMDIKKINDG LLDSKILGAF  50
                     4.3bits
       Sequence logo         MMADSKLVSL NNNLSGKIKD QGKVIKNYYG TMDIKKINDG LLDSKILGAF
                     0.0bits
                                      60                        80                       100
                                       |                         |                         |
gi|29432477|gb|AAC83193.2|   NTVIALLGSI IIIVMNIMII QNYTRTTDNQ ALIKESLQSV QQQIKALTDK 100
gi|328495173|gb|AEB21198.1|  NTVIALLGSI IIIVMNIMII QNYTRTTDNQ ALIKESLQSV QQQIKALTDK 100
gi|328495213|gb|AEB21234.1|  NTVIALLGSI IIIVMNIMII QNYTRTTDNQ ALIKESLQSV QQQIKALTDK 100
gi|350998758|gb|AEQ38062.1|  NTVIALLGSI IIIVMNIMII QNYTRTTDNQ ALIKESLQSV QQQIKALTDK 100
gi|350998768|gb|AEQ38071.1|  NTVIALLGSI IIIVMNIMII QNYTRTTDNQ ALIKESLQSV QQQIKALTDK 100
gi|350998778|gb|AEQ38080.1|  NTVIALLGSI IIIVMNIMII QNYTRTTDNQ ALIKESLQSV QQQIKALTDK 100
gi|350998842|gb|AEQ38133.1|  NTVIALLGSI IIIVMNIMII QNYTRTTDNQ ALIKESLQSV QQQIKALTDK 100
gi|350998851|gb|AEQ38141.1|  NTVIALLGSI IIIVMNIMII QNYTRTTDNQ ALIKESLQSV QQQIKALTDK 100
gi|29468607|ref|NP_047112.2| NTVIALLGSI IIIVMNIMII QNYTRTTDNQ ALIKESLQSV QQQIKALTDK 100
gi|328495182|gb|AEB21206.1|  NTVIALLGSI IIIVMNIMII QNYTRTTDNQ ALIKESLQSV QQQIKALTDK 100
gi|328495203|gb|AEB21225.1|  NTVIALLGSI IIIVMNIMII QNYTRTTDNQ ALIKESLQSV QQQIKALTDK 100
gi|350998831|gb|AEQ38124.1|  NTVIALLGSI IIIVMNIMII QNYTRTTDNQ ALIKESLQSV QQQIKALTDK 100
gi|350998718|gb|AEQ38026.1|  NTVIALLGSI IIIVMNIMII QNYTRTTDNQ ALIKESLQSV QQQIKALTDK 100
gi|350998728|gb|AEQ38035.1|  NTVIALLGSI IIIVMNIMII QNYTRTTDNQ ALIKESLQSV QQQIKALTDK 100
gi|350998737|gb|AEQ38043.1|  NTVIALLGSI IIIVMNIMII QNYTRTTDNQ ALIKESLQSV QQQIKALTDK 100
gi|350998747|gb|AEQ38052.1|  NTVIALLGSI IIIVMNIMII QNYTRTTDNQ ALIKESLQSV QQQIKALTDK 100
gi|350998803|gb|AEQ38099.1|  NTVIALLGSI IIIVMNIMII QNYTRTTDNQ ALIKESLQSV QQQIKALTDK 100
gi|350998821|gb|AEQ38115.1|  NTVIALLGSI IIIVMNIMII QNYTRTTDNQ ALIKESLQSV QQQIKALTDK 100
gi|350998813|gb|AEQ38108.1|  NTVIALLGSI IIIVMNIMII QNYTRTTDNQ ALIKESLQSV QQQIKALTDK 100
gi|328495193|gb|AEB21216.1|  NTVIALLGSI IIIVMNIMII QNYTRTTDNQ ALIKESLQSV QQQIKALTDK 100
                     4.3bits
       Sequence logo         NTVIALLGSI IIIVMNIMII QNYTRTTDNQ ALIKESLQSV QQQIKALTDK
                     0.0bits
                                                           120                       140
                                                             |                         |
gi|29432477|gb|AAC83193.2|   IGTEIGPKVS LIDTSSTITI PANIGLLGSK ISQSTSSINE NVNDKCKFTL 150
gi|328495173|gb|AEB21198.1|  IGTEIGPKVS LIDTSSTITI PANIGLLGSK ISQSTSSINE NVNDKCKFTL 150
gi|328495213|gb|AEB21234.1|  IGTEIGPKVS LIDTSSTITI PANIGLLGSK ISQSTSSINE NVNDKCKFTL 150
gi|350998758|gb|AEQ38062.1|  IGTEIGPKVS LIDTSSTITI PANIGLLGSK ISQSTSSINE NVNDKCKFTL 150
gi|350998768|gb|AEQ38071.1|  IGTEIGPKVS LIDTSSTITI PANIGLLGSK ISQSTSSINE NVNDKCKFTL 150
gi|350998778|gb|AEQ38080.1|  IGTEIGPKVS LIDTSSTITI PANIGLLGSK ISQSTSSINE NVNDKCKFTL 150
gi|350998842|gb|AEQ38133.1|  IGTEIGPKVS LIDTSSTITI PANIGLLGSK ISQSTSSINE NVNDKCKFTL 150
gi|350998851|gb|AEQ38141.1|  IGTEIGPKVS LIDTSSTITI PANIGLLGSK ISQSTSSINE NVNDKCKFTL 150
gi|29468607|ref|NP_047112.2| IGTEIGPKVS LIDTSSTITI PANIGLLGSK ISQSTSSINE NVNDKCKFTL 150
gi|328495182|gb|AEB21206.1|  IGTEIGPKVS LIDTSSTITI PANIGLLGSK ISQSTSSINE NVNDKCKFTL 150
gi|328495203|gb|AEB21225.1|  IGTEIGPKVS LIDTSSTITI PANIGLLGSK ISQSTSSINE NVNDKCKFTL 150
gi|350998831|gb|AEQ38124.1|  IGTEIGPKVS LIDTSSTITI PANIGLLGSK ISQSTSSINE NVNDKCKFTL 150
gi|350998718|gb|AEQ38026.1|  IGTEIGPKVS LIDTSSTITI PANIGLLGSK ISQSTSSINE NVNDKCKFTL 150
gi|350998728|gb|AEQ38035.1|  IGTEIGPKVS LIDTSSTITI PANIGLLGSK ISQSTSSINE NVNDKCKFTL 150
gi|350998737|gb|AEQ38043.1|  IGTEIGPKVS LIDTSSTITI PANIGLLGSK ISQSTSSINE NVNDKCKFTL 150
gi|350998747|gb|AEQ38052.1|  IGTEIGPKVS LIDTSSTITI PANIGLLGSK ISQSTSSINE NVNDKCKFTL 150
gi|350998803|gb|AEQ38099.1|  IGTEIGPKVS LIDTSSTITI PANIGLLGSK ISQSTSSINE NVNDKCKFTL 150
gi|350998821|gb|AEQ38115.1|  IGTEIGPKVS LIDTSSTITI PANIGLLGSK ISQSTSSINE NVNDKCKFTL 150
gi|350998813|gb|AEQ38108.1|  IGTEIGPKVS LIDTSSTITI PANIGLLGSK ISQSTSSINE NVNDKCKFTL 150
gi|328495193|gb|AEB21216.1|  IGTEIGPKVS LIDTSSTITI PANIGLLGSK ISQSTSSINE NVNDKCKFTL 150
                     4.3bits
       Sequence logo         IGTEIGPKVS LIDTSSTITI PANIGLLGSK ISQSTSSINE NVNDKCKFTL
                     0.0bits
```

	160		180		200	
gi|29432477|gb|AAC83193.2|	PPLKIHECNI	SCPNPLPFRE	YRPISQGVSD	LVGLPNQICL	QKTTSTILKP	200
gi|328495173|gb|AEB21198.1|	PPLKIHECNI	SCPNPLPFRE	YRPISQGVSD	LVGLPNQICL	QKTTSTILKP	200
gi|328495213|gb|AEB21234.1|	PPLKIHECNI	SCPNPLPFRE	YRPISQGVSD	LVGLPNQICL	QKTTSTILKP	200
gi|350998758|gb|AEQ38062.1|	PPLKIHECNI	SCPNPLPFRE	YRPISQGVSD	LVGLPNQICL	QKTTSTILKP	200
gi|350998768|gb|AEQ38071.1|	PPLKIHECNI	SCPNPLPFRE	YRPISQGVSD	LVGLPNQICL	QKTTSTILKP	200
gi|350998778|gb|AEQ38080.1|	PPLKIHECNI	SCPNPLPFRE	YRPISQGVSD	LVGLPNQICL	QKTTSTILKP	200
gi|350998842|gb|AEQ38133.1|	PPLKIHECNI	SCPNPLPFRE	YRPISQGVSD	LVGLPNQICL	QKTTSTILKP	200
gi|350998851|gb|AEQ38141.1|	PPLKIHECNI	SCPNPLPFRE	YRPISQGVSD	LVGLPNQICL	QKTTSTILKP	200
gi|29468607|ref|NP_047112.2|	PPLKIHECNI	SCPNPLPFRE	YRPISQGVSD	LVGLPNQICL	QKTTSTILKP	200
gi|328495182|gb|AEB21206.1|	PPLKIHECNI	SCPNPLPFRE	YRPISQGVSD	LVGLPNQICL	QKTTSTILKP	200
gi|328495203|gb|AEB21225.1|	PPLKIHECNI	SCPNPLPFRE	YRPISQGVSD	LVGLPNQICL	QKTTSTILKP	200
gi|350998831|gb|AEQ38124.1|	PPLKIHECNI	SCPNPLPFRE	YRPISQGVSD	LVGLPNQICL	QKTTSTILKP	200
gi|350998718|gb|AEQ38026.1|	PPLKIHECNI	SCPNPLPFRE	YRPISQGVSD	LVGLPNQICL	QKTTSTILKP	200
gi|350998728|gb|AEQ38035.1|	PPLKIHECNI	SCPNPLPFRE	YRPISQGVSD	LVGLPNQICL	QKTTSTILKP	200
gi|350998737|gb|AEQ38043.1|	PPLKIHECNI	SCPNPLPFRE	YRPISQGVSD	LVGLPNQICL	QKTTSTILKP	200
gi|350998747|gb|AEQ38052.1|	PPLKIHECNI	SCPNPLPFRE	YRPISQGVSD	LVGLPNQICL	QKTTSTILKP	200
gi|350998803|gb|AEQ38099.1|	PPLKIHECNI	SCPNPLPFRE	YRPISQGVSD	LVGLPNQICL	QKTTSTILKP	200
gi|350998821|gb|AEQ38115.1|	PPLKIHECNI	SCPNPLPFRE	YRPISQGVSD	LVGLPNQICL	QKTTSTILKP	200
gi|350998813|gb|AEQ38108.1|	PPLKIHECNI	SCPNPLPFRE	YRPISQGVSD	LVGLPNQICL	QKTTSTILKP	200
gi|328495193|gb|AEB21216.1|	PPLKIHECNI	SCPNPLPFRE	YRPISQGVSD	LVGLPNQICL	QKTTSTILKP	200

Sequence logo 4.3bits 0.0bits

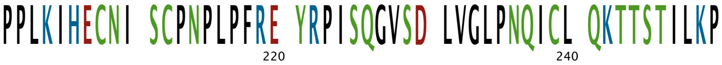

		220		240		
gi|29432477|gb|AAC83193.2|	RLISYTLPIN	TREGVCITDP	LLAVDNGFFA	YSHLEKIGSC	TRGIAKQRII	250
gi|328495173|gb|AEB21198.1|	RLISYTLPIN	TREGVCITDP	LLAVDNGFFA	YSHLEKIGSC	TRGIAKQRII	250
gi|328495213|gb|AEB21234.1|	RLISYTLPIN	TREGVCITDP	LLAVDNGFFA	YSHLEKIGSC	TRGIAKQRII	250
gi|350998758|gb|AEQ38062.1|	RLISYTLPIN	TREGVCITDP	LLAVDNGFFA	YSHLEKIGSC	TRGIAKQRII	250
gi|350998768|gb|AEQ38071.1|	RLISYTLPIN	TREGVCITDP	LLAVDNGFFA	YSHLEKIGSC	TRGIAKQRII	250
gi|350998778|gb|AEQ38080.1|	RLISYTLPIN	TREGVCITDP	LLAVDNGFFA	YSHLEKIGSC	TRGIAKQRII	250
gi|350998842|gb|AEQ38133.1|	RLISYTLPIN	TREGVCITDP	LLAVDNGFFA	YSHLEKIGSC	TRGIAKQRII	250
gi|350998851|gb|AEQ38141.1|	RLISYTLPIN	TREGVCITDP	LLAVDNGFFA	YSHLEKIGSC	TRGIAKQRII	250
gi|29468607|ref|NP_047112.2|	RLISYTLPIN	TREGVCITDP	LLAVDNGFFA	YSHLEKIGSC	TRGIAKQRII	250
gi|328495182|gb|AEB21206.1|	RLISYTLPIN	TREGVCITDP	LLAVDNGFFA	YSHLEKIGSC	TRGIAKQRII	250
gi|328495203|gb|AEB21225.1|	RLISYTLPIN	TREGVCITDP	LLAVDNGFFA	YSHLEKIGSC	TRGIAKQRII	250
gi|350998831|gb|AEQ38124.1|	RLISYTLPIN	TREGVCITDP	LLAVDNGFFA	YSHLEKIGSC	TRGIAKQRII	250
gi|350998718|gb|AEQ38026.1|	RLISYTLPIN	TREGVCITDP	LLAVDNGFFA	YSHLEKIGSC	TRGIAKQRII	250
gi|350998728|gb|AEQ38035.1|	RLISYTLPIN	TREGVCITDP	LLAVDNGFFA	YSHLEKIGSC	TRGIAKQRII	250
gi|350998737|gb|AEQ38043.1|	RLISYTLPIN	TREGVCITDP	LLAVDNGFFA	YSHLEKIGSC	TRGIAKQRII	250
gi|350998747|gb|AEQ38052.1|	RLISYTLPIN	TREGVCITDP	LLAVDNGFFA	YSHLEKIGSC	TRGIAKQRII	250
gi|350998803|gb|AEQ38099.1|	RLISYTLPIN	TREGVCITDP	LLAVDNGFFA	YSHLEKIGSC	TRGIAKQRII	250
gi|350998821|gb|AEQ38115.1|	RLISYTLPIN	TREGVCITDP	LLAVDNGFFA	YSHLEKIGSC	TRGIAKQRII	250
gi|350998813|gb|AEQ38108.1|	RLISYTLPIN	TREGVCITDP	LLAVDNGFFA	YSHLEKIGSC	TRGIAKQRII	250
gi|328495193|gb|AEB21216.1|	RLISYTLPIN	TREGVCITDP	LLAVDNGFFA	YSHLEKIGSC	TRGIAKQRII	250

Sequence logo 4.3bits 0.0bits

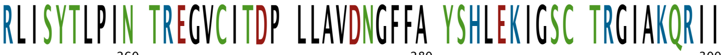

	260		280		300	
gi|29432477|gb|AAC83193.2|	GVGEVLDRGD	KVPSMFMTNV	WTPPNPSTIH	HCSSTYHEDF	YYTLCAVSHV	300
gi|328495173|gb|AEB21198.1|	GVGEVLDRGD	KVPSMFMTNV	WTPPNPSTIH	HCSSTYHEDF	YYTLCAVSHV	300
gi|328495213|gb|AEB21234.1|	GVGEVLDRGD	KVPSMFMTNV	WTPPNPSTIH	HCSSTYHEDF	YYTLCAVSHV	300
gi|350998758|gb|AEQ38062.1|	GVGEVLDRGD	KVPSMFMTNV	WTPPNPSTIH	HCSSTYHEDF	YYTLCAVSHV	300
gi|350998768|gb|AEQ38071.1|	GVGEVLDRGD	KVPSMFMTNV	WTPPNPSTIH	HCSSTYHEDF	YYTLCAVSHV	300
gi|350998778|gb|AEQ38080.1|	GVGEVLDRGD	KVPSMFMTNV	WTPPNPSTIH	HCSSTYHEDF	YYTLCAVSHV	300
gi|350998842|gb|AEQ38133.1|	GVGEVLDRGD	KVPSMFMTNV	WTPPNPSTIH	HCSSTYHEDF	YYTLCAVSHV	300
gi|350998851|gb|AEQ38141.1|	GVGEVLDRGD	KVPSMFMTNV	WTPPNPSTIH	HCSSTYHEDF	YYTLCAVSHV	300
gi|29468607|ref|NP_047112.2|	GVGEVLDRGD	KVPSMFMTNV	WTPPNPSTIH	HCSSTYHEDF	YYTLCAVSHV	300
gi|328495182|gb|AEB21206.1|	GVGEVLDRGD	KVPSMFMTNV	WTPPNPSTIH	HCSSTYHEDF	YYTLCAVSHV	300
gi|328495203|gb|AEB21225.1|	GVGEVLDRGD	KVPSMFMTNV	WTPPNPSTIH	HCSSTYHEDF	YYTLCAVSHV	300
gi|350998831|gb|AEQ38124.1|	GVGEVLDRGD	KVPSMFMTNV	WTPPNPSTIH	HCSSTYHEDF	YYTLCAVSHV	300
gi|350998718|gb|AEQ38026.1|	GVGEVLDRGD	KVPSMFMTNV	WTPPNPSTIH	HCSSTYHEDF	YYTLCAVSHV	300
gi|350998728|gb|AEQ38035.1|	GVGEVLDRGD	KVPSMFMTNV	WTPPNPSTIH	HCSSTYHEDF	YYTLCAVSHV	300
gi|350998737|gb|AEQ38043.1|	GVGEVLDRGD	KVPSMFMTNV	WTPPNPSTIH	HCSSTYHEDF	YYTLCAVSHV	300
gi|350998747|gb|AEQ38052.1|	GVGEVLDRGD	KVPSMFMTNV	WTPPNPSTIH	HCSSTYHEDF	YYTLCAVSHV	300
gi|350998803|gb|AEQ38099.1|	GVGEVLDRGD	KVPSMFMTNV	WTPPNPSTIH	HCSSTYHEDF	YYTLCAVSHV	300
gi|350998821|gb|AEQ38115.1|	GVGEVLDRGD	KVPSMFMTNV	WTPPNPSTIH	HCSSTYHEDF	YYTLCAVSHV	300
gi|350998813|gb|AEQ38108.1|	GVGEVLDRGD	KVPSMFMTNV	WTPPNPSTIH	HCSSTYHEDF	YYTLCAVSHV	300
gi|328495193|gb|AEB21216.1|	GVGEVLDRGD	KVPSMFMTNV	WTPPNPSTIH	HCSSTYHEDF	YYTLCAVSHV	300

Sequence logo 4.3bits 0.0bits

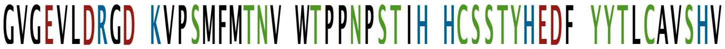

```
                                                     320                           340
                                                       |                             |
gi|29432477|gb|AAC83193.2|   GDPILNSTSW TESLSLIRLA VRPKSDSGDY NQKYIAITKV ERGKYDKVMP 350
gi|328495173|gb|AEB21198.1|  GDPILNSTSW TESLSLIRLA VRPKSDSGDY NQKYIAITKV ERGKYDKVMP 350
gi|328495213|gb|AEB21234.1|  GDPILNSTSW TESLSLIRLA VRPKSDSGDY NQKYIAITKV ERGKYDKVMP 350
gi|350998758|gb|AEQ38062.1|  GDPILNSTSW TESLSLIRLA VRPKSDSGDY NQKYIAITKV ERGKYDKVMP 350
gi|350998768|gb|AEQ38071.1|  GDPILNSTSW TESLSLIRLA VRPKSDSGDY NQKYIAITKV ERGKYDKVMP 350
gi|350998778|gb|AEQ38080.1|  GDPILNSTSW TESLSLIRLA VRPKSDSGDY NQKYIAITKV ERGKYDKVMP 350
gi|350998842|gb|AEQ38133.1|  GDPILNSTSW TESLSLIRLA VRPKSDSGDY NQKYIAITKV ERGKYDKVMP 350
gi|350998851|gb|AEQ38141.1|  GDPILNSTSW TESLSLIRLA VRPKSDSGDY NQKYIAITKV ERGKYDKVMP 350
gi|29468607|ref|NP_047112.2| GDPILNSTSW TESLSLIRLA VRPKSDSGDY NQKYIAITKV ERGKYDKVMP 350
gi|328495182|gb|AEB21206.1|  GDPILNSTSW TESLSLIRLA VRPKSDSGNY NQKYIAITKV ERGKYDKVMP 350
gi|328495203|gb|AEB21225.1|  GDPILNSTSW TESLSLIRLA VRPKSDSGDY NQKYIAITKV ERGKYDKVMP 350
gi|350998831|gb|AEQ38124.1|  GDPILNSTSW TESLSLIRLA VRPKSDSGDY NQKYIAITKV ERGKYDKVMP 350
gi|350998718|gb|AEQ38026.1|  GDPILNSTSW TESLSLIRLA VRPKSDSGDY NQKYITITKV ERGKYDKVMP 350
gi|350998728|gb|AEQ38035.1|  GDPILNSTSW TESLSLIRLA VRPKSDSGDY NQKYITITKV ERGKYDKVMP 350
gi|350998737|gb|AEQ38043.1|  GDPILNSTSW TESLSLIRLA VRPKSDSGDY NQKYITITKV ERGKYDKVMP 350
gi|350998747|gb|AEQ38052.1|  GDPILNSTSW TESLSLIRLA VRPKSDSGDY NQKYITITKV ERGKYDKVMP 350
gi|350998803|gb|AEQ38099.1|  GDPILNSTSW TESLSLIRLA VRPKSDSGDY NQKYITITKV ERGKYDKVMP 350
gi|350998821|gb|AEQ38115.1|  GDPILNSTSW TESLSLIRLA VRPKSDNGDY NQKYIAITKV ERGKYDKVMP 350
gi|350998813|gb|AEQ38108.1|  GDPILNSTSW TESLSLIRLA VRPKSDSGDY NQKYITITKV ERGKYDKVMP 350
gi|328495193|gb|AEB21216.1|  GDPILNSTSW TESLSLIRLA VRPKSDSGDY NQKYIAINKV ERGKYDKVMP 350
                     4.3bits
       Sequence logo         GDPILNSTSW TESLSLIRLA VRPKSDSGDY NQKYIAITKV ERGKYDKVMP
                     0.0bits
                                    360                  380                  400
                                      |                    |                    |
gi|29432477|gb|AAC83193.2|   YGPSGIKQGD TLYFPAVGFL PRTEFQYNDS NCPIIHCKYS KAENCRLSMG 400
gi|328495173|gb|AEB21198.1|  YGPSGIKQGD TLYFPAVGFL PRTEFQYNDS NCPIIHCKYS KAENCRLSMG 400
gi|328495213|gb|AEB21234.1|  YGPSGIKQGD TLYFPAVGFL PRTEFQYNDS NCPIIHCKYS KAENCRLSMG 400
gi|350998758|gb|AEQ38062.1|  YGPSGIKQGD TLYFPAVGFL PRTEFQYNDS NCPIIHCKYS KAENCRLSMG 400
gi|350998768|gb|AEQ38071.1|  YGPSGIKQGD TLYFPAVGFL PRTEFQYNDS NCPIIHCKYS KAENCRLSMG 400
gi|350998778|gb|AEQ38080.1|  YGPSGIKQGD TLYFPAVGFL PRTEFQYNDS NCPIIHCKYS KAENCRLSMG 400
gi|350998842|gb|AEQ38133.1|  YGPSGIKQGD TLYFPAVGFL PRTEFQYNDS NCPIIHCKYS KAENCRLSMG 400
gi|350998851|gb|AEQ38141.1|  YGPSGIKQGD TLYFPAVGFL PRTEFQYNDS NCPIIHCKYS KAENCRLSMG 400
gi|29468607|ref|NP_047112.2| YGPSGIKQGD TLYFPAVGFL PRTEFQYNDS NCPIIHCKYS KAENCRLSMG 400
gi|328495182|gb|AEB21206.1|  YGPSGIKQGD TLYFPAVGFL PRTEFQYNDS NCPIIHCKYS KAENCRLSMG 400
gi|328495203|gb|AEB21225.1|  YGPSGIKQGD TLYFPAVGFL PRTEFQYNDS NCPIIHCKYS KAENCRLSMG 400
gi|350998831|gb|AEQ38124.1|  YGPSGIKQGD TLYFPAVGFL PRTEFQYNDS NCPIIHCKYS KAENCRLSMG 400
gi|350998718|gb|AEQ38026.1|  YGPSGIKQGD TLYFPAVGFL PRTEFQYNDS NCPIIHCKYS KAENCRLSMG 400
gi|350998728|gb|AEQ38035.1|  YGPSGIKQGD TLYFPAVGFL PRTEFQYNDS NCPIIHCKYS KAENCRLSMG 400
gi|350998737|gb|AEQ38043.1|  YGPSGIKQGD TLYFPAVGFL PRTEFQYNDS NCPIIHCKYS KAENCRLSMG 400
gi|350998747|gb|AEQ38052.1|  YGPSGIKQGD TLYFPAVGFL PRTEFQYNDS NCPIIHCKYS KAENCRLSMG 400
gi|350998803|gb|AEQ38099.1|  YGPSGIKQGD TLYFPAVGFL PRTEFQYNDS NCPIIHCKYS KAENCRLSMG 400
gi|350998821|gb|AEQ38115.1|  YGPSGIKQGD TLYFPAVGFL PRTEFQYNDS NCPIIHCKYS KAENCRLSMG 400
gi|350998813|gb|AEQ38108.1|  YGPSGIKQGN TLYFPAVGFL PRTEFQYNDS NCPIIHCKYS KAENCRLSMG 400
gi|328495193|gb|AEB21216.1|  YGPSGIKQGD TLYFPAVGFL PRTEFQYNDS NCPIIHCKYS KAENCRLSMG 400
                     4.3bits
       Sequence logo         YGPSGIKQGD TLYFPAVGFL PRTEFQYNDS NCPIIHCKYS KAENCRLSMG
                     0.0bits
                                                     420                  440
                                                       |                    |
gi|29432477|gb|AAC83193.2|   VNSKSHYILR SGLLKYNLSL GGDIILQFIE IADNRLTIGS PSKIYNSLGQ 450
gi|328495173|gb|AEB21198.1|  VNSKSHYILR SGLLKYNLSL GGDIILQFIE IADNRLTIGS PSKIYNSLGQ 450
gi|328495213|gb|AEB21234.1|  VNSKSHYILR SGLLKYNLSL GGDIILQFIE IADNRLTIGS PSKIYNSLGQ 450
gi|350998758|gb|AEQ38062.1|  VNSKSHYILR SGLLKYNLSL GGDIILQFIE IADNRLTIGS PSKIYNSLGQ 450
gi|350998768|gb|AEQ38071.1|  VNSKSHYILR SGLLKYNLSL GGDIILQFIE IADNRLTIGS PSKIYNSLGQ 450
gi|350998778|gb|AEQ38080.1|  VNSKSHYILR SGLLKYNLSL GGDIILQFIE IADNRLTIGS PSKIYNSLGQ 450
gi|350998842|gb|AEQ38133.1|  VNSKSHYILR SGLLKYNLSL GGDIILQFIE IADNRLTIGS PSKIYNSLGQ 450
gi|350998851|gb|AEQ38141.1|  VNSKSHYILR SGLLKYNLSL GGDIILQFIE IADNRLTIGS PSKIYNSLGQ 450
gi|29468607|ref|NP_047112.2| VNSKSHYILR SGLLKYNLSL GGDIILQFIE IADNRLTIGS PSKIYNSLGQ 450
gi|328495182|gb|AEB21206.1|  VNSKSHYILR SGLLKYNLSL GGDIILQFIE IADNRLTIGS PSKIYNSLGQ 450
gi|328495203|gb|AEB21225.1|  VNSKSHYILR SGLLKYNLSL GGDITLQFIE IADNRLTIGS PSKIYNSLGQ 450
gi|350998831|gb|AEQ38124.1|  VNSKSHYILR SGLLKYNLSL GGDITLQFIE IADNRLTIGS PSKIYNSLGQ 450
gi|350998718|gb|AEQ38026.1|  VNSKSHYILR SGLLKYNLSL GGDIILQFIE IADNRLTIGS PSKIYNSLGQ 450
gi|350998728|gb|AEQ38035.1|  VNSKSHYILR SGLLKYNLSL GGDIILQFIE IADNRLTIGS PSKIYNSLGQ 450
gi|350998737|gb|AEQ38043.1|  VNSKSHYILR SGLLKYNLSL GGDIILQFIE IADNRLTIGS PSKIYNSLGQ 450
gi|350998747|gb|AEQ38052.1|  VNSKSHYILR SGLLKYNLSL GGDIILQFIE IADNRLTIGS PSKIYNSLGQ 450
gi|350998803|gb|AEQ38099.1|  VNSKSHYILR SGLLKYNLSL GGDIILQFIE IADNRLTIGS PSKIYNSLGQ 450
gi|350998821|gb|AEQ38115.1|  VNSKSHYILR SGLLKYNLSL GGDIILQFIE IADNRLTIGS PSKIYNSLGQ 450
gi|350998813|gb|AEQ38108.1|  VNSKSHYILR SGLLKYNLSL GGDIILQFIE IADNRLTIGS PSKIYNSLGQ 450
gi|328495193|gb|AEB21216.1|  VNSKSHYILR SGLLKYNLSL GGDIILQFIE IADNRLTIGS PSKIYNSLGQ 450
                     4.3bits
       Sequence logo         VNSKSHYILR SGLLKYNLSL GGDIILQFIE IADNRLTIGS PSKIYNSLGQ
                     0.0bits
```

```
                                   460                            480                            500
                                    |                              |                              |
gi|29432477|gb|AAC83193.2|   PVFYQASYSW DTMIKLGDVD TVDPLRVQWR NNSVISRPGQ SQCPRFNVCP 500
gi|328495173|gb|AEB21198.1|  PVFYQASYSW DTMIKLGDVD TVDPLRVQWR NNSVISRPGQ SQCPRFNVCP 500
gi|328495213|gb|AEB21234.1|  PVFYQASYSW DTMIKLGDVD TVDPLRVQWR NNSVISRPGQ SQCPRFNVCP 500
gi|350998758|gb|AEQ38062.1|  PVFYQASYSW DTMIKLGDVD TVDPLRVQWR NNSVISRPGQ SQCPRFNVCP 500
gi|350998768|gb|AEQ38071.1|  PVFYQASYSW DTMIKLGDVD TVDPLRVQWR NNSVISRPGQ SQCPRFNVCP 500
gi|350998778|gb|AEQ38080.1|  PVFYQASYSW DTMIKLGDVD TVDPLRVQWR NNSVISRPGQ SQCPRFNVCP 500
gi|350998842|gb|AEQ38133.1|  PVFYQASYSW DTMIKLGDVD TVDPLRVQWR NNSVISRPGQ SQCPRFNVCP 500
gi|350998851|gb|AEQ38141.1|  PVFYQASYSW DTMIKLGDVD TVDPLRVQWR NNSVISRPGQ SQCPRFNVCP 500
gi|29468607|ref|NP_047112.2| PVFYQASYSW DTMIKLGDVD TVDPLRVQWR NNSVISRPGQ SQCPRFNVCP 500
gi|328495182|gb|AEB21206.1|  PVFYQASYSW DTMIKLGDID TVDPLRVQWR NNSVISRPGQ SQCPRFNVCP 500
gi|328495203|gb|AEB21225.1|  PVFYQASYSW DTMIKLGDVD TVDPLRVQWR NNSVISRPGQ SQCPRFNVCP 500
gi|350998831|gb|AEQ38124.1|  PVFYQASYSW DTMIKLGDVD TVDPLRVQWR NNSVISRPGQ SQCPRFNVCP 500
gi|350998718|gb|AEQ38026.1|  PVFYQASYSW DTMIKLGDVD TVDPLRVQWR NNSVISRPGQ SQCPRFNVCP 500
gi|350998728|gb|AEQ38035.1|  PVFYQASYSW DTMIKLGDVD TVDPLRVQWR NNSVISRPGQ SQCPRFNVCP 500
gi|350998737|gb|AEQ38043.1|  PVFYQASYSW DTMIKLGDVD TVDPLRVQWR NNSVISRPGQ SQCPRFNVCP 500
gi|350998747|gb|AEQ38052.1|  PVFYQASYSW DTMIKLGDVD TVDPLRVQWR NNSVISRPGQ SQCPRFNVCP 500
gi|350998803|gb|AEQ38099.1|  PVFYQASYSW DTMIKLGDVD TVDPLRVQWR NNSVISRPGQ SQCPRFNVCP 500
gi|350998821|gb|AEQ38115.1|  PVFYQASYSW DTMIKLGDVD TVDPLRVQWR NNSVISRPGQ SQCPRFNVCP 500
gi|350998813|gb|AEQ38108.1|  PVFYQASYSW DTMIKLGDVD TVDPLRVQWR NNSVISRPGQ SQCPRFNVCP 500
gi|328495193|gb|AEB21216.1|  PVFYQASYSW DTMIKLGDVD TVDPLRVQWR NNSVISRPGQ SQCPRFNVCP 500
                     4.3bits
       Sequence logo         PVFYQASYSW DTMIKLGDVD TVDPLRVQWR NNSVISRPGQ SQCPRFNVCP
                     0.0bits
                                                  520                            540
                                                   |                              |
gi|29432477|gb|AAC83193.2|   EVCWEGTYND AFLIDRLNWV SAGVYLNSNQ TAENPVFAVF KDNEILYQVP 550
gi|328495173|gb|AEB21198.1|  EVCWEGTYND AFLIDRLNWV SAGVYLNSNQ TAENPVFAVF KDNEILYQVP 550
gi|328495213|gb|AEB21234.1|  EVCWEGTYND AFLIDRLNWV SAGVYLNSNQ TAENPVFAVF KDNEILYQVP 550
gi|350998758|gb|AEQ38062.1|  EVCWEGTYND AFLIDRLNWV SAGVYLNSNQ TAENPVFAVF KDNEILYQVP 550
gi|350998768|gb|AEQ38071.1|  EVCWEGTYND AFLIDRLNWV SAGVYLNSNQ TAENPVFAVF KDNEILYQVP 550
gi|350998778|gb|AEQ38080.1|  EVCWEGTYND AFLIDRLNWV SAGVYLNSNQ TAENPVFAVF KDNEILYQVP 550
gi|350998842|gb|AEQ38133.1|  EVCWEGTYND AFLIDRLNWV SAGVYLNSNQ TAENPVFAVF KDNEILYQVP 550
gi|350998851|gb|AEQ38141.1|  EVCWEGTYND AFLIDRLNWV SAGVYLNSNQ TAENPVFAVF KDNEILYQVP 550
gi|29468607|ref|NP_047112.2| EVCWEGTYND AFLIDRLNWV SAGVYLNSNQ TAENPVFAVF KDNEILYQVP 550
gi|328495182|gb|AEB21206.1|  EVCWEGTYND AFLIDRLNWV SAGVYLNSNQ TAENPVFAVF KDNEILYQVP 550
gi|328495203|gb|AEB21225.1|  EVCWEGTYND AFLIDRLNWV SAGVYLNSNQ TAENPVFAVF KDNEILYQVP 550
gi|350998831|gb|AEQ38124.1|  EVCWEGTYND AFLIDRLNWV SAGVYLNSNQ TAENPVFAVF KDNEILYQVP 550
gi|350998718|gb|AEQ38026.1|  EVCWEGTYND AFLIDRLNWV SAGVYLNSNQ TAENPVFAVF KDNEILYQVP 550
gi|350998728|gb|AEQ38035.1|  EVCWEGTYND AFLIDRLNWV SAGVYLNSNQ TAENPVFAVF KDNEILYQVP 550
gi|350998737|gb|AEQ38043.1|  EVCWEGTYND AFLIDRLNWV SAGVYLNSNQ TAENPVFAVF KDNEILYQVP 550
gi|350998747|gb|AEQ38052.1|  EVCWEGTYND AFLIDRLNWV SAGVYLNSNQ TAENPVFAVF KDNEILYQVP 550
gi|350998803|gb|AEQ38099.1|  EVCWEGTYND AFLIDRLNWV SAGVYLNSNQ TAENPVFAVF KDNEILYQVP 550
gi|350998821|gb|AEQ38115.1|  EVCWEGTYND AFLIDRLNWV SAGVYLNSNQ TAENPVFAVF KDNEILYQVP 550
gi|350998813|gb|AEQ38108.1|  EVCWEGTYND AFLIDRLNWV SAGVYLNSNQ TAENPVFAVF KDNEILYQVP 550
gi|328495193|gb|AEB21216.1|  EVCWEGTYND AFLIDRLNWV SAGVYLNSNQ TAENPVFAVF KDNEILYQVP 550
                     4.3bits
       Sequence logo         EVCWEGTYND AFLIDRLNWV SAGVYLNSNQ TAENPVFAVF KDNEILYQVP
                     0.0bits
                                   560                            580                            600
                                    |                              |                              |
gi|29432477|gb|AAC83193.2|   LAEDDTNAQK TITDCFLLEN VIWCISLVEI YDTGDSVIRP KLFAVKIPAQ 600
gi|328495173|gb|AEB21198.1|  LAEDDTNAQK TITDCFLLEN VIWCISLVEI YDTGDSVIRP KLFAVKIPAQ 600
gi|328495213|gb|AEB21234.1|  LAEDDTNAQK TITDCFLLEN VIWCISLVEI YDTGDSVIRP KLFAVKIPAQ 600
gi|350998758|gb|AEQ38062.1|  LAEDDTNAQK TITDCFLLEN VIWCISLVEI YDTGDSVIRP KLFAVKIPAQ 600
gi|350998768|gb|AEQ38071.1|  LAEDDTNAQK TITDCFLLEN VIWCISLVEI YDTGDSVIRP KLFAVKIPAQ 600
gi|350998778|gb|AEQ38080.1|  LAEDDTNAQK TITDCFLLEN VIWCISLVEI YDTGDSVIRP KLFAVKIPAQ 600
gi|350998842|gb|AEQ38133.1|  LAEDDTNAQK TITDCFLLEN VIWCISLVEI YDTGDSVIRP KLFAVKIPAQ 600
gi|350998851|gb|AEQ38141.1|  LAEDDTNAQK TITDCFLLEN VIWCISLVEI YDTGDSVIRP KLFAVKIPAQ 600
gi|29468607|ref|NP_047112.2| LAEDDTNAQK TITDCFLLEN VIWCISLVEI YDTGDSVIRP KLFAVKIPAQ 600
gi|328495182|gb|AEB21206.1|  LAEDDTNAQK TITDCFLLEN VIWCISLVEI YDTGDSVIRP KLFAVKIPAQ 600
gi|328495203|gb|AEB21225.1|  LAEDDTNAQK TITDCFLLEN VIWCISLVEI YDTGDSVIRP KLFAVKIPAQ 600
gi|350998831|gb|AEQ38124.1|  LAEDDTNAQK TITDCFLLEN VIWCISLVEI YDTGDSVIRP KLFAVKIPAQ 600
gi|350998718|gb|AEQ38026.1|  LAEDDTNAQK TITDCFLLEN VIWCISLVEI YDTGDSVIRP KLFAVKIPAQ 600
gi|350998728|gb|AEQ38035.1|  LAEDDTNAQK TITDCFLLEN VIWCISLVEI YDTGDSVIRP KLFAVKIPAQ 600
gi|350998737|gb|AEQ38043.1|  LAEDDTNAQK TITDCFLLEN VIWCISLVEI YDTGDSVIRP KLFAVKIPAQ 600
gi|350998747|gb|AEQ38052.1|  LAEDDTNAQK TITDCFLLEN VIWCISLVEI YDTGDSVIRP KLFAVKIPAQ 600
gi|350998803|gb|AEQ38099.1|  LAEDDTNAQK TITDCFLLEN VIWCISLVEI YDTGDSVIRP KLFAVKIPAQ 600
gi|350998821|gb|AEQ38115.1|  LAEDDTNAQK TITDCFLLEN VIWCISLVEI YDTGDSVIRP KLFAVKIPAQ 600
gi|350998813|gb|AEQ38108.1|  LAEDDTNAQK TITDCFLLEN VIWCISLVEI YDTGDSVIRP KLFAVKIPAQ 600
gi|328495193|gb|AEB21216.1|  LAEDDTNAQK TITDCFLLEN VIWCISLVEI YDTGDSVIRP KLFAVKIPAQ 600
                     4.3bits
       Sequence logo         LAEDDTNAQK TITDCFLLEN VIWCISLVEI YDTGDSVIRP KLFAVKIPAQ
                     0.0bits
```

gi|29432477|gb|AAC83193.2|	C S E S	604
gi|328495173|gb|AEB21198.1|	C S E S	604
gi|328495213|gb|AEB21234.1|	C S E S	604
gi|350998758|gb|AEQ38062.1|	C S E S	604
gi|350998768|gb|AEQ38071.1|	C S E S	604
gi|350998778|gb|AEQ38080.1|	C S E S	604
gi|350998842|gb|AEQ38133.1|	C S E S	604
gi|350998851|gb|AEQ38141.1|	C S E S	604
gi|29468607|ref|NP_047112.2|	C S E S	604
gi|328495182|gb|AEB21206.1|	C S E S	604
gi|328495203|gb|AEB21225.1|	C S E S	604
gi|350998831|gb|AEQ38124.1|	C S E S	604
gi|350998718|gb|AEQ38026.1|	C S E S	604
gi|350998728|gb|AEQ38035.1|	C S E S	604
gi|350998737|gb|AEQ38043.1|	C S E S	604
gi|350998747|gb|AEQ38052.1|	C S E S	604
gi|350998803|gb|AEQ38099.1|	C S E S	604
gi|350998821|gb|AEQ38115.1|	C S E S	604
gi|350998813|gb|AEQ38108.1|	C S E S	604
gi|328495193|gb|AEB21216.1|	C S E S	604

4.3bits
Sequence logo
0.0bits

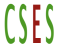

Supplement: Report S2 — Alignment of G proteins in all reported Hendra virus isolates in Genebank. (PDF) [file ppat.1003684.s009.pdf]
