# Supplementary material for: Crystal Structure of the Hendra Virus Attachment G Glycoprotein Bound to a Potent Cross-Reactive Neutralizing Human Monoclonal Antibody
Source: PLoS Pathog. 2013 Oct 10;9(10):e1003684. doi: 10.1371/journal.ppat.1003684 (PMC3795035; doi:10.1371/journal.ppat.1003684)

```
                                                           20                   40
                                                            |                    |
gi|13518009|gb|AAK29088.1|    MPAENKKVRF ENTTSDKGKI PSKVIKSYYG TMDIKKINEG LLDSKILSAF 50
gi|15487369|gb|AAK50545.1|    MPAENKKVRF ENTTSDKGKI PSKVIKSYYG TMDIKKINEG LLDSKILSAF 50
gi|15487379|gb|AAK50554.1|    MPAENKKVRF ENTTSDKGKI PSKVIKSYYG TMDIKKINEG LLDSKILSAF 50
gi|20152868|gb|AAM13406.1|    MPAENKKVRF ENTTSDKGKI PSKVIKSYYG TMDIKKINEG LLDSKILSAF 50
gi|40644703|emb|CAD92351.1|   MPAENKKVRF ENTTSDKGKI PSKVIKSYYG TMDIKKINEG LLDSKILSAF 50
gi|40644710|emb|CAD92357.1|   MPAENKKVRF ENTTSDKGKI PSKVIKSYYG TMDIKKINEG LLDSKILSAF 50
gi|40644717|emb|CAD92363.1|   MPAENKKVRF ENTTSDKGKI PSKVIKSYYG TMDIKKINEG LLDSKILSAF 50
gi|13559814|ref|NP_112027.1|  MPAENKKVRF ENTTSDKGKI PSKVIKSYYG TMDIKKINEG LLDSKILSAF 50
gi|57282813|emb|CAF25497.1|   MPAENKKVRF ENTTSDKGKN PSKVIKSYYG TMDIKKINEG LLDSKILSAF 50
gi|307557085|gb|ADN51995.1|   MPAENKKVRF ENTTSDKGKN PSKVIKSYYG TMDIKKINEG LLDSKILSAF 50
gi|313476293|emb|CBM41034.1|  MPAENKKVRF ENTTSDKGKN PSKVIKSYYG TMDIKKINEG LLDSKILSAF 50
gi|66271898|gb|AAY43916.1|    MPTESKKVRF ENTASDKGKN PSKVIKSYYG TMDIKKINEG LLDSKILSAF 50
gi|374256950|gb|AEZ01382.1|   MPTESKKVRF ENTASDKGKN PSKVIKSYYG TMDIKKINEG LLDSKILSAF 50
gi|374256958|gb|AEZ01389.1|   MPTESKKVRF ENTASDKGKN PSKVIKSYYG TMDIKKINEG LLDSKILSAF 50
gi|253559848|gb|ACT32615.1|   MPTESKKVRF ENTASDKGKN PSKVIKSYYG TMDIKKINEG LLDSKILSAF 50
gi|374256967|gb|AEZ01397.1|   MPTESKKVRF ENTASDKGKN PSKVIKSYYG TMDIKKINEG LLDSKILSAF 50
gi|357640965|gb|AET87354.1|   ---------- ---------- ---------- ---------- ---------- -
                      4.3bits
Sequence logo
                      0.0bits
```
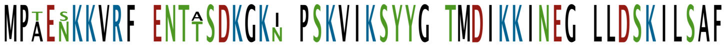

```
                                                60                   80                   100
                                                 |                    |                    |
gi|13518009|gb|AAK29088.1|    NTVIALLGSI VIIVMNIMII QNYTRSTDNQ AVIKDALQGI QQQIKGLADK 100
gi|15487369|gb|AAK50545.1|    NTVIALLGSI VIIVMNIMII QNYTRSTDNQ AVIKDALQGI QQQIKGLADK 100
gi|15487379|gb|AAK50554.1|    NTVIALLGSI VIIVMNIMII QNYTRSTDNQ AVIKDALQGI QQQIKGLADK 100
gi|20152868|gb|AAM13406.1|    NTVIALLGSI VIIVMNIMII QNYTRSTDNQ AVIKDALQGI QQQIKGLADK 100
gi|40644703|emb|CAD92351.1|   NTVIALLGSI VIIVMNIMII QNYTRSTDNQ AVIKDALQGI QQQIKGLADK 100
gi|40644710|emb|CAD92357.1|   NTVIALLGSI VIIVMNIMII QNYTRSTDNQ AVIKDALQGI QQQIKGLADK 100
gi|40644717|emb|CAD92363.1|   NTVIALLGSI VIIVMNIMII QNYTRSTDNQ AVIKDALQGI QQQIKGLADK 100
gi|13559814|ref|NP_112027.1|  NTVIALLGSI VIIVMNIMII QNYTRSTDNQ AVIKDALQGI QQQIKGLADK 100
gi|57282813|emb|CAF25497.1|   NTVIALLGSI VIIVMNIMII QNYTRSTDNQ AVIKDALQGI QQQIKGLADK 100
gi|307557085|gb|ADN51995.1|   NTVIALLGSI VIIVMNIMII QNYTRSTDNQ AVIKDALQGI QQQIKGLADK 100
gi|313476293|emb|CBM41034.1|  NTVIALLGSI VIIVMNIMII QNYTRSTDNQ AVIKDALQGI QQQIKGLADK 100
gi|66271898|gb|AAY43916.1|    NTVIALLGSI VIIVMNIMII QNYTRSTDNQ AMIKDALQSI QQQIKGLADK 100
gi|374256950|gb|AEZ01382.1|   NTVIALLGSI VIIVMNIMII QNYTRSTDNQ AMIKDALQSI QQQIKGLADK 100
gi|374256958|gb|AEZ01389.1|   NTVIALLGSI VIIVMNIMII QNYTRSTDNQ AMIKDALQSI QQQIKGLADK 100
gi|253559848|gb|ACT32615.1|   NTVIALLGSI VIIVMNIMII QNYTRSTDNQ AMIKDALQSI QQQIKGLADK 100
gi|374256967|gb|AEZ01397.1|   NTVIALLGSI VIIVMNIMII QNYTRSTDNQ AMIKDALQSI QQQIKGLADK 100
gi|357640965|gb|AET87354.1|   ---------- ---------- ---------- ---------- ---------- -
                      4.3bits
Sequence logo
                      0.0bits
```
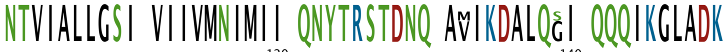

```
                                                           120                  140
                                                            |                    |
gi|13518009|gb|AAK29088.1|    IGTEIGPKVS LIDTSSTITI PANIGLLGSK ISQSTASINE NVNEKCKFTL 150
gi|15487369|gb|AAK50545.1|    IGTEIGPKVS LIDTSSTITI PANIGLLGSK ISQSTASINE NVNEKCKFTL 150
gi|15487379|gb|AAK50554.1|    IGTEIGPKVS LIDTSSTITI PANIGLLGSK ISQSTASINE NVNEKCKFTL 150
gi|20152868|gb|AAM13406.1|    IGTEIGPKVS LIDTSSTITI PANIGLLGSK ISQSTASINE NVNEKCKFTL 150
gi|40644703|emb|CAD92351.1|   IGTEIGPKVS LIDTSSTITI PANIGLLGSK ISQSTASINE NVNEKCKFTL 150
gi|40644710|emb|CAD92357.1|   IGTEIGPKVS LIDTSSTITI PANIGLLGSK ISQSTASINE NVNEKCKFTL 150
gi|40644717|emb|CAD92363.1|   IGTEIGPKVS LIDTSSTITI PANIGLLGSK ISQSTASINE NVNEKCKFTL 150
gi|13559814|ref|NP_112027.1|  IGTEIGPKVS LIDTSSTITI PANIGLLGSK ISQSTASINE NVNEKCKFTL 150
gi|57282813|emb|CAF25497.1|   IGTEIGPKVS LIDTSSTITI PANIGLLGSK ISQSTASINE NVNEKCKFTL 150
gi|307557085|gb|ADN51995.1|   IGTEIGPKVS LIDTSSTITI PANIGLLGSK ISQSTASINE NVNEKCKFTL 150
gi|313476293|emb|CBM41034.1|  IGTEIGPKVS LIDTSSTITI PANIGLLGSK ISQSTASINE NVNEKCKFTL 150
gi|66271898|gb|AAY43916.1|    IGTEIGPKVS LIDTSSTITI PANIGLLGSK ISQSTASINE NVNEKCKFTL 150
gi|374256950|gb|AEZ01382.1|   IGTEIGPKVS LIDTSSTITI PANIGLLGSK ISQSTASINE NVNEKCKFTL 150
gi|374256958|gb|AEZ01389.1|   IGTEIGPKVS LIDTSSTITI PANIGLLGSK ISQSTASINE NVNEKCKFTL 150
gi|253559848|gb|ACT32615.1|   IGTEIGPKVS LIDTSSTITI PANIGLLGSK ISQSTASINE NVNEKCKFTL 150
gi|374256967|gb|AEZ01397.1|   IGTEIGPKVS LIDTSSTITI PANIGLLGSK ISQSTASINE NVNEKCKFTL 150
gi|357640965|gb|AET87354.1|   ---------- ---------- ---------- ---------- ---------- -
                      4.3bits
Sequence logo
                      0.0bits
```
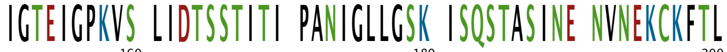

```
                                                160                  180                  200
                                                 |                    |                    |
gi|13518009|gb|AAK29088.1|    PPLKIHECNI SCPNPLPFRE YRPQTEGVSN LVGLPNNICL QKTSNQILKP 200
gi|15487369|gb|AAK50545.1|    PPLKIHECNI SCPNPLPFRE YRPQTEGVSN LVGLPNNICL QKTSNQILKP 200
gi|15487379|gb|AAK50554.1|    PPLKIHECNI SCPNPLPFRE YRPQTEGVSN LVGLPNNICL QKTSNQILKP 200
gi|20152868|gb|AAM13406.1|    PPLKIHECNI SCPNPLPFRE YRPQTEGVSN LVGLPNNICL QKTSNQILKP 200
gi|40644703|emb|CAD92351.1|   PPLKIHECNI SCPNPLPFRE YRPQTEGVSN LVGLPNNICL QKTSNQILKP 200
gi|40644710|emb|CAD92357.1|   PPLKIHECNI SCPNPLPFRE YRPQTEGVSN LVGLPNNICL QKTSNQILKP 200
gi|40644717|emb|CAD92363.1|   PPLKIHECNI SCPNPLPFRE YRPQTEGVSN LVGLPNNICL QKTSNQILKP 200
gi|13559814|ref|NP_112027.1|  PPLKIHECNI SCPNPLPFRE YRPQTEGVSN LVGLPNNICL QKTSNQILKP 200
gi|57282813|emb|CAF25497.1|   PPLKIHECNI SCPNPLPFRE YRPQTEGVSN LVGLPNNICL QKTSNQILKP 200
gi|307557085|gb|ADN51995.1|   PPLKIHECNI SCPNPLPFRE YRPQTEGVSN LVGLPDNICL QKTSNQILKP 200
gi|313476293|emb|CBM41034.1|  PPLKIHECNI SCPNPLPFRE YRPQTEGVSN LVGLPDNICL QKTSNQILKP 200
gi|66271898|gb|AAY43916.1|    PPLKIHECNI SCPNPLPFRE YKPQTEGVSN LVGLPNNICL QKTSNQILKP 200
gi|374256950|gb|AEZ01382.1|   PPLKIHECNI SCPNPLPFRE YKPQTEGVSN LVGLPNNICL QKTSNQILKP 200
gi|374256958|gb|AEZ01389.1|   PPLKIHECNI SCPNPLPFRE YKPQTEGVSN LVGLPNNICL QKTSNQILKP 200
gi|253559848|gb|ACT32615.1|   PPLKIHECNI SCPNPLPFRE YKPQTEGVSN LVGLPNNICL QKTSNQILKP 200
gi|374256967|gb|AEZ01397.1|   PPLKIHECNI SCPNPLPFRE YKPQTEGVSN LVGLPNNICL QKTSNQILKP 200
gi|357640965|gb|AET87354.1|   ---------- ---------- ---------- ---------- ---------- -
                      4.3bits
Sequence logo
                      0.0bits
```
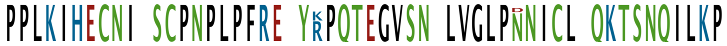

```
                                          220                           240
                                            |                             |
gi|13518009|gb|AAK29088.1|    KLISYTLPVV GQSGTCITDP LLAMDEGYFA YSHLERIGSC SRGVSKQRII 250
gi|15487369|gb|AAK50545.1|    KLISYTLPVV GQSGTCITDP LLAMDEGYFA YSHLERIGSC SRGVSKQRII 250
gi|15487379|gb|AAK50554.1|    KLISYTLPVV GQSGTCITDP LLAMDEGYFA YSHLERIGSC SRGVSKQRII 250
gi|20152868|gb|AAM13406.1|    KLISYTLPVV GQSGTCITDP LLAMDEGYFA YSHLERIGSC SRGVSKQRII 250
gi|40644703|emb|CAD92351.1|   KLISYTLPVV GQSGTCITDP LLAMDEGYFA YSHLERIGSC SRGVSKQRII 250
gi|40644710|emb|CAD92357.1|   KLISYTLPVV GQSGTCITDP LLAMDEGYFA YSHLERIGSC SRGVSKQRII 250
gi|40644717|emb|CAD92363.1|   KLISYTLPVV GQSGTCITDP LLAMDEGYFA YSHLERIGSC SRGVSKQRII 250
gi|13559814|ref|NP_112027.1|  KLISYTLPVV GQSGTCITDP LLAMDEGYFA YSHLERIGSC SRGVSKQRII 250
gi|57282813|emb|CAF25497.1|   KLISYTLPVV GQSGTCITDP LLAMDEGYFA YSHLERIGSC SRGVSKQRII 250
gi|307557085|gb|ADN51995.1|   KLISYTLPVV GQSGTCITDP LLAMDEGYFA YSHLERIGSC SRGVSKQRII 250
gi|313476293|emb|CBM41034.1|  KLISYTLPVV GQSGTCITDP LLAMDEGYFA YSHLERIGSC SRGVSKQRII 250
gi|66271898|gb|AAY43916.1|    KLISYTLPVV GQSGTCITDP LLAMDEGYFA YSHLEKIGSC SRGVSKQRII 250
gi|374256950|gb|AEZ01382.1|   KLISYTLPVV GQSGTCITDP LLAMDEGYFA YSHLEKIGSC SRGVSKQRII 250
gi|374256958|gb|AEZ01389.1|   KLISYTLPVV GQSGTCITDP LLAMDEGYFA YSHLEKIGSC SRGVSKQRII 250
gi|253559848|gb|ACT32615.1|   KLISYTLPVV GQSGTCITDP LLAMDEGYFA YSHLEKIGSC SRGVSKQRII 250
gi|374256967|gb|AEZ01397.1|   KLISYTLPVV GQSGTCITDP LLAMDEGYFA YSHLEKIGSC SRGVSKQRII 250
gi|357640965|gb|AET87354.1|   ---------- ---------- -------YFA YSHLEKIGSC SRGVSKQRII 23
```

Sequence logo

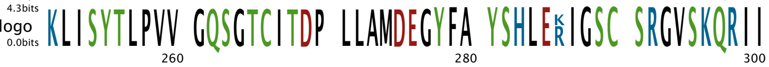

```
                                    260                           280                           300
                                      |                             |                             |
gi|13518009|gb|AAK29088.1|    GVGEVLDRGD EVPSLFMTNV WTPPNPNTVY HCSAVYNNEF YYVLCAVSTV 300
gi|15487369|gb|AAK50545.1|    GVGEVLDRGD EVPSLFMTNV WTPPNPNTVY HCSAVYNNEF YYVLCAVSTV 300
gi|15487379|gb|AAK50554.1|    GVGEVLDRGD EVPSLFMTNV WTPPNPNTVY HCSAVYNNEF YYVLCAVSTV 300
gi|20152868|gb|AAM13406.1|    GVGEVLDRGD EVPSLFMTNV WTPPNPNTVY HCSAVYNNEF YYVLCAVSTV 300
gi|40644703|emb|CAD92351.1|   GVGEVLDRGD EVPSLFMTNV WTPPNPNTVY HCSAVYNNEF YYVLCAVSTV 300
gi|40644710|emb|CAD92357.1|   GVGEVLDRGD EVPSLFMTNV WTPPNPNTVY HCSAVYNNEF YYVLCAVSTV 300
gi|40644717|emb|CAD92363.1|   GVGEVLDRGD EVPSLFMTNV WTPPNPNTVY HCSAVYNNEF YYVLCAVSTV 300
gi|13559814|ref|NP_112027.1|  GVGEVLDRGD EVPSLFMTNV WTPPNPNTVY HCSAVYNNEF YYVLCAVSTV 300
gi|57282813|emb|CAF25497.1|   GVGEVLDRGD EVPSLFMTNV WAPPNPNTVY HCSAVYNNEF YYVLCAVSTV 300
gi|307557085|gb|ADN51995.1|   GVGEVLDRGD EVPSLFMTNV WTPPNPNTVY HCSAVYNNEF YYVLCAVSTV 300
gi|313476293|emb|CBM41034.1|  GVGEVLDRGD EVPSLFMTNV WTPPNPNTVY HCSAVYNNEF YYVLCAVSTV 300
gi|66271898|gb|AAY43916.1|    GVGEVLDRGD EVPSLFMTNV WTPSNPNTVY HCSAVYNNEF YYVLCAVSVV 300
gi|374256950|gb|AEZ01382.1|   GVGEVLDRGD EVPSLFMTNV WTPSNPNTVY HCSAVYNNEF YYVLCAVSVV 300
gi|374256958|gb|AEZ01389.1|   GVGEVLDRGD EVPSLFMTNV WTPSNPNTVY HCSAVYNNEF YYVLCAVSVV 300
gi|253559848|gb|ACT32615.1|   GVGEVLDRGD EVPSLFMTNV WTPSNPNTVY HCSAVYNNEF YYVLCAVSVV 300
gi|374256967|gb|AEZ01397.1|   GVGEVLDRGD EVPSLFMTNV WTPSNPNTVY HCSAVYNNEF YYVLCAVSVV 300
gi|357640965|gb|AET87354.1|   GVGEVLDRGD EVPSLFMTNV WTPSNPNTVY HCSAVYNNEF YYVLCAVSVV 73
```

Sequence logo

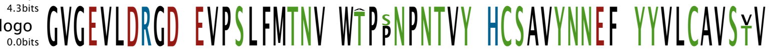

```
                                                          320                           340
                                                            |                             |
gi|13518009|gb|AAK29088.1|    GDPILNSTYW SGSLMMTRLA VKPKSNGGGY NQHQLALRSI EKGRYDKVMP 350
gi|15487369|gb|AAK50545.1|    GDPILNSTYW SGSLMMTRLA VKPKSNGGGY NQHQLALRSI EKGRYDKVMP 350
gi|15487379|gb|AAK50554.1|    GDPILNSTYW SGSLMMTRLA VKPKSNGGGY NQHQLALRSI EKGRYDKVMP 350
gi|20152868|gb|AAM13406.1|    GDPILNSTYW SGSLMMTRLA VKPKSNGGGY NQHQLALRSI EKGRYDKVMP 350
gi|40644703|emb|CAD92351.1|   GDPILNSTYW SGSLMMTRLA VKPKSNGGGY NQHQLALRSI EKGRYDKVMP 350
gi|40644710|emb|CAD92357.1|   GDPILNSTYW SGSLMMTRLA VKPKSNGGGY NQHQLALRSI EKGRYDKVMP 350
gi|40644717|emb|CAD92363.1|   GDPILNSTYW SGSLMMTRLA VKPKSNGGGY NQHQLALRSI EKGRYDKVMP 350
gi|13559814|ref|NP_112027.1|  GDPILNSTYW SGSLMMTRLA VKPKSNGGGY NQHQLALRSI EKGRYDKVMP 350
gi|57282813|emb|CAF25497.1|   GDPILNSTYW SGSLMMTRLA VKPKSNGGGY NQHQLALRSI EKGRYDKVMP 350
gi|307557085|gb|ADN51995.1|   GDPILNSTYW SGSLMMTRLA VKPKSNGGGY NQHQLALRSI EKGRYDKVMP 350
gi|313476293|emb|CBM41034.1|  GDPILNSTYW SGSLMMTRLA VKPKSNGGGY NQHQLALRSI EKGRYDKVMP 350
gi|66271898|gb|AAY43916.1|    GDPILNSTYW SGSLMMTRLA VKPKNNGESY NQHQFALRNI EKGKYDKVMP 350
gi|374256950|gb|AEZ01382.1|   GDPILNSTYW SGSLMMTRLA VKPKNNGESY NQHQFALRNI EKGKYDKVMP 350
gi|374256958|gb|AEZ01389.1|   GDPILNSTYW SGSLMMTRLA VKPKNNGESY NQHQFALRNI EKGKYDKVMP 350
gi|253559848|gb|ACT32615.1|   GDPVLNSTYW SGSLMMTRLA VKPKNNGESY NQHQFALRNI EKGKYDKVMP 350
gi|374256967|gb|AEZ01397.1|   GDPILNSTYW SGSLMMTRLA VKPKNNGESY NQHQFALRNI EKGKYDKVMP 350
gi|357640965|gb|AET87354.1|   GDPILNSTYW SGSLMMTRLA VKPKNNGESY NQHQFALRNI EKGKYDKVMP 123
```

Sequence logo

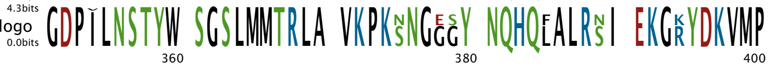

```
                                    360                           380                           400
                                      |                             |                             |
gi|13518009|gb|AAK29088.1|    YGPSGIKQGD TLYFPAVGFL VRTEFKYNDS NCPITKCQYS KPENCRLSMG 400
gi|15487369|gb|AAK50545.1|    YGPSGIKQGD TLYFPAVGFL VRTEFKYNDS NCPITKCQYS KPENCRLSMG 400
gi|15487379|gb|AAK50554.1|    YGPSGIKQGD TLYFPAVGFL VRTEFKYNDS NCPITKCQYS KPENCRLSMG 400
gi|20152868|gb|AAM13406.1|    YGPSGIKQGD TLYFPAVGFL VRTEFKYNDS NCPITKCQYS KPENCRLSMG 400
gi|40644703|emb|CAD92351.1|   YGPSGIKQGD TLYFPAVGFL VRTEFKYNDS NCPITKCQYS KPENCRLSMG 400
gi|40644710|emb|CAD92357.1|   YGPSGIKQGD TLYFPAVGFL VRTEFKYNDS NCPITKCQYS KPENCRLSMG 400
gi|40644717|emb|CAD92363.1|   YGPSGIKQGD TLYFPAVGFL VRTEFKYNDS NCPITKCQYS KPENCRLSMG 400
gi|13559814|ref|NP_112027.1|  YGPSGIKQGD TLYFPAVGFL VRTEFKYNDS NCPITKCQYS KPENCRLSMG 400
gi|57282813|emb|CAF25497.1|   YGPSGIKQGD TLYFPAVGFL VRTEFKYNDS NCPITKCQYS KPENCRLSMG 400
gi|307557085|gb|ADN51995.1|   YGPSGIKQGD TLYFPAVGFL VRTEFKYNDS NCPITKCQYS KPENCRLSMG 400
gi|313476293|emb|CBM41034.1|  YGPSGIKQGD TLYFPAVGFL VRTEFKYNDS NCPITKCQYS KPENCRLSMG 400
gi|66271898|gb|AAY43916.1|    YGPSGIKQGD TLYFPAVGFL VRTEFKYNDS NCPIAECQYS KPENCRLSMG 400
gi|374256950|gb|AEZ01382.1|   YGPSGIKQGD TLYFPAVGFL VRTEFKYNDS NCPIAECQYS KPENCRLSMG 400
gi|374256958|gb|AEZ01389.1|   YGPSGIKQGD TLYFPAVGFL VRTEFKYNDS NCPIAECQYS KPENCRLSMG 400
gi|253559848|gb|ACT32615.1|   YGPSGIKQGD TLYFPAVGFL VRTEFKYNDS NCPIAECQYS KPENCRLSMG 400
gi|374256967|gb|AEZ01397.1|   YGPSGIKQGD TLYFPAVGFL VRTEFTYNDS NCPIAECQYS KPENCRLSMG 400
gi|357640965|gb|AET87354.1|   YGPSGIKQGD TLYFPAVGFL VRTEFTYNDS NCPIAECQYS KPENCRLSMG 173
```

Sequence logo

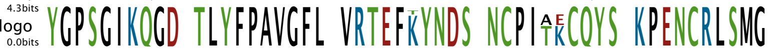

```
                                                420                  440
                                                 |                    |
gi|13518009|gb|AAK29088.1|    IRPNSHYILR SGLLKYNLSD GENPKVVFIE ISDQRLSIGS PSKIYDSLGQ 450
gi|15487369|gb|AAK50545.1|    IRPNSHYILR SGLLKYNLSD GENPKVVFIE ISDQRLSIGS PSKIYDSLGQ 450
gi|15487379|gb|AAK50554.1|    IRPNSHYILR SGLLKYNLSD GENPKVVFIE ISDQRLSIGS PSKIYDSLGQ 450
gi|20152868|gb|AAM13406.1|    IRPNSHYILR SGLLKYNLSD GENPKVVFIE ISDQRLSIGS PSKIYDSLGQ 450
gi|40644703|emb|CAD92351.1|   IRPNSHYILR SGLLKYNLSD GENPKVVFIE ISDQRLSIGS PSKIYDSLGQ 450
gi|40644710|emb|CAD92357.1|   IRPNSHYILR SGLLKYNLSD GENPKVVFIE ISDQRLSIGS PSKIYDSLGQ 450
gi|40644717|emb|CAD92363.1|   IRPNSHYILR SGLLKYNLSD GENPKVVFIE ISDQRLSIGS PSKIYDSLGQ 450
gi|13559814|ref|NP_112027.1|  IRPNSHYILR SGLLKYNLSD GENPKVVFIE ISDQRLSIGS PSKIYDSLGQ 450
gi|57282813|emb|CAF25497.1|   IRPNSHYILR SGLLKYNLSD GENPKVVFIE ISDQRLSIGS PSKIYDSLGQ 450
gi|307557085|gb|ADN51995.1|   IRPNSHYILR SGLLKYNLSD GENPKIVFIE ISDQRLSIGS PSKVYDSLGQ 450
gi|313476293|emb|CBM41034.1|  IRPNSHYILR SGLLKYNLSD GENPKIVFIE ISDQRLSIGS PSKVYDSLGQ 450
gi|66271898|gb|AAY43916.1|    IRPNSHYILR SGLLKYNLSD EENSKIVFIE ISDQRLSIGS PSKIYDSLGQ 450
gi|374256950|gb|AEZ01382.1|   IRPNSHYILR SGLLKYNLSD EENSKIVFIE ISDQRLSIGS PSKIYDSLGQ 450
gi|374256958|gb|AEZ01389.1|   IRPNSHYILR SGLLKYNLSD EENSKIVFIE ISDQRLSIGS PSKIYDSLGQ 450
gi|253559848|gb|ACT32615.1|   IRPNSHYILR SGLLKYNLSD EENSKIVFIE ISDQRLSIGS PSKIYDSLGQ 450
gi|374256967|gb|AEZ01397.1|   IRPNSHYILR SGLLKYNLSD EENSKIVFIE ISDQRLSIGS PSKIYDSLGQ 450
gi|357640965|gb|AET87354.1|   IRPNSHYILR SGLLKYNLSD EENSKIVFIE ISDQRLSIGS PSKIYDSLGQ 223
```

Sequence logo (4.3bits / 0.0bits)

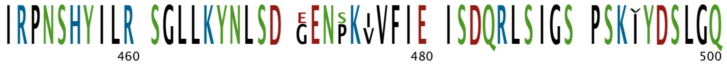

```
                                   460                  480                  500
                                    |                    |                    |
gi|13518009|gb|AAK29088.1|    PVFYQASFSW DTMIKFGDVL TVNPLVVNWR NNTVISRPGQ SQCPRFNTCP 500
gi|15487369|gb|AAK50545.1|    PVFYQASFSW DTMIKFGDVL TVNPLVVNWR NNTVISRPGQ SQCPRFNTCP 500
gi|15487379|gb|AAK50554.1|    PVFYQASFSW DTMIKFGDVL TVNPLVVNWR NNTVISRPGQ SQCPRFNTCP 500
gi|20152868|gb|AAM13406.1|    PVFYQASFSW DTMIKFGDVL TVNPLVVNWR NNTVISRPGQ SQCPRFNTCP 500
gi|40644703|emb|CAD92351.1|   PVFYQASFSW DTMIKFGDVL TVNPLVVNWR NNTVISRPGQ SQCPRFNTCP 500
gi|40644710|emb|CAD92357.1|   PVFYQASFSW DTMIKFGDVL TVNPLVVNWR NNTVISRPGQ SQCPRFNTCP 500
gi|40644717|emb|CAD92363.1|   PVFYQASFSW DTMIKFGDVL TVNPLVVNWR NNTVISRPGQ SQCPRFNTCP 500
gi|13559814|ref|NP_112027.1|  PVFYQASFSW DTMIKFGDVL TVNPLVVNWR NNTVISRPGQ SQCPRFNTCP 500
gi|57282813|emb|CAF25497.1|   PVFYQASFSW DTMIKFGDVL TVNPLVVNWR NNTVISRPGQ SQCPRFNTCP 500
gi|307557085|gb|ADN51995.1|   PVFYQASFSW DTMIKFGDVQ TVNPLVVNWR DNTVISRPGQ SQCPRFNTCP 500
gi|313476293|emb|CBM41034.1|  PVFYQASFSW DTMIKFGDVQ TVNPLVVNWR DNTVISRPGQ SQCPRFNTCP 500
gi|66271898|gb|AAY43916.1|    PVFYQASFSW DTMIKFGDVQ TVNPLVVNWR DNTVISRPGQ SQCPRFNKCP 500
gi|374256950|gb|AEZ01382.1|   PVFYQASFSW DTMIKFGDVQ TVNPLVVNWR DNTVISRPGQ SQCPRFNKCP 500
gi|374256958|gb|AEZ01389.1|   PVFYQASFSW DTMIKFGDVQ TVNPLVVNWR DNTVISRPGQ SQCPRFNKCP 500
gi|253559848|gb|ACT32615.1|   PVFYQASFSW DTMIKFGDVQ TVNPLVVNWR DNTVISRPGQ SQCPRFNKCP 500
gi|374256967|gb|AEZ01397.1|   PVFYQASFSW DTMIKFGDVQ TVNPLVVNWR DNTVISRPGQ SQCPRFNKCP 500
gi|357640965|gb|AET87354.1|   PVFYQASFSW DTMIKFGDVQ TVNPLVVNWR DNTVISRPGQ SQCPRFNKCP 273
```

Sequence logo (4.3bits / 0.0bits)

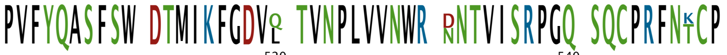

```
                                                520                  540
                                                 |                    |
gi|13518009|gb|AAK29088.1|    EICWEGVYND AFLIDRINWI SAGVFLDSNQ TAENPVFTVF KDNEILYRAQ 550
gi|15487369|gb|AAK50545.1|    EICWEGVYND AFLIDRINWI SAGVFLDSNQ TAENPVFTVF KDNEILYRAQ 550
gi|15487379|gb|AAK50554.1|    EICWEGVYND AFLIDRINWI SAGVFLDSNQ TAENPVFTVF KDNEILYRAQ 550
gi|20152868|gb|AAM13406.1|    EICWEGVYND AFLIDRINWI SAGVFLDSNQ TAENPVFTVF KDNEILYRAQ 550
gi|40644703|emb|CAD92351.1|   EICWEGVYND AFLIDRINWI SAGVFLDSNQ TAENPVFTVF KDNEILYRAQ 550
gi|40644710|emb|CAD92357.1|   EICWEGVYND AFLIDRINWI SAGVFLDSNQ TAENPVFTVF KDNEILYRAQ 550
gi|40644717|emb|CAD92363.1|   EICWEGVYND AFLIDRINWI SAGVFLDSNQ TAENPVFTVF KDNEILYRAQ 550
gi|13559814|ref|NP_112027.1|  EICWEGVYND AFLIDRINWI SAGVFLDSNQ TAENPVFTVF KDNEILYRAQ 550
gi|57282813|emb|CAF25497.1|   EICWEGVYND AFLIDRINWI SAGVFLDSNQ TAENPVFTVF KDNEILYRAQ 550
gi|307557085|gb|ADN51995.1|   EICWEGVYND AFLIDRINWI SAGVFLDSNQ TAENPVFTVF KDNEILYRAQ 550
gi|313476293|emb|CBM41034.1|  EICWEGVYND AFLIDRINWI SAGVFLDSNQ TAENPVFTVF KDNEILYRAQ 550
gi|66271898|gb|AAY43916.1|    EVCWEGVYND AFLIDRINWI SAGVFLDSNQ TAENPVFTVF KDNEVLYRAQ 550
gi|374256950|gb|AEZ01382.1|   EVCWEGVYND AFLIDRINWI SAGVFLDSNQ TAENPVFTVF KDNEVLYRAQ 550
gi|374256958|gb|AEZ01389.1|   EVCWEGVYND AFLIDRINWI SAGVFLDSNQ TAENPVFTVF KDNEVLYRAQ 550
gi|253559848|gb|ACT32615.1|   EVCWEGVYND AFLIDRINWI SAGVFLDSNQ TAENPVFTVF KDNEVLYRAQ 550
gi|374256967|gb|AEZ01397.1|   EVCWEGVYND AFLIDRINWI SAGVFLDSNQ TAENPVFTVF KDNEVLYRAQ 550
gi|357640965|gb|AET87354.1|   EVCWEGVYND AFLIDRINWI SAGVFLDSNQ TAENPVFTVF KDNEV----- 318
```

Sequence logo (4.3bits / 0.0bits)

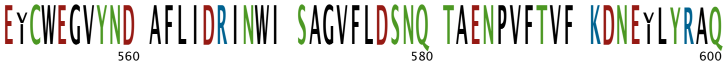

```
                                   560                  580                  600
                                    |                    |                    |
gi|13518009|gb|AAK29088.1|    LASEDTNAQK TITNCFLLKN KIWCISLVEI YDTGDNVIRP KLFAVKIPEQ 600
gi|15487369|gb|AAK50545.1|    LASEDTNAQK TITNCFLLKN KIWCISLVEI YDTGDNVIRP KLFAVKIPEQ 600
gi|15487379|gb|AAK50554.1|    LASEDTNAQK TITNCFLLKN KIWCISLVEI YDTGDNVIRP KLFAVKIPEQ 600
gi|20152868|gb|AAM13406.1|    LASEDTNAQK TITNCFLLKN KIWCISLVEI YDTGDNVIRP KLFAVKIPEQ 600
gi|40644703|emb|CAD92351.1|   LASEDTNAQK TITNCFLLKN KIWCISLVEI YDTGDNVIRP KLFAVKIPEQ 600
gi|40644710|emb|CAD92357.1|   LASEDTNAQK TITNCFLLKN KIWCISLVEI YDTGDNVIRP KLFAVKIPEQ 600
gi|40644717|emb|CAD92363.1|   LASEDTNAQK TITNCFLLKN KIWCISLVEI YDTGDNVIRP KLFAVKIPEQ 600
gi|13559814|ref|NP_112027.1|  LASEDTNAQK TITNCFLLKN KIWCISLVEI YDTGDNVIRP KLFAVKIPEQ 600
gi|57282813|emb|CAF25497.1|   LASEDTNAQK TITNCFLLKN KIWCISLVEI YDTGDNVIRP KLFAVKIPEQ 600
gi|307557085|gb|ADN51995.1|   LASEDTNAQK TITNCFLLKN KIWCISLVEI YDTGDNVIRP KLFAVKIPEQ 600
gi|313476293|emb|CBM41034.1|  LASEDTNAQK TITNCFLLKN KIWCISLVEI YDTGDNVIRP KLFAVKIPEQ 600
gi|66271898|gb|AAY43916.1|    LASEDTNAQK TITNCFLLKN KIWCISLVEI YDTGDNVIRP KLFAVKIPEQ 600
gi|374256950|gb|AEZ01382.1|   LASEDTNAQK TITNCFLLKN KIWCISLVEI YDTGDNVIRP KLFAVKIPEQ 600
gi|374256958|gb|AEZ01389.1|   LASEDTNAQK TITNCFLLKN KIWCISLVEI YDTGDNVIRP KLFAVKIPEQ 600
gi|253559848|gb|ACT32615.1|   LASEDTNAQK TITNCFLLKN KIWCISLVEI YDTGDNVIRP KLFAVKIPEQ 600
gi|374256967|gb|AEZ01397.1|   LASEDTNAQK TITNCFLLKN KIWCISLVEI YDTGDNVIRP KLFAVKIPEQ 600
gi|357640965|gb|AET87354.1|   ---------- ---------- ---------- ---------- ---------- 318
```

Sequence logo (4.3bits / 0.0bits)

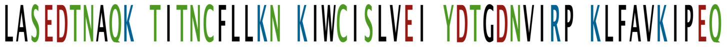

0.0bits

gi|13518009|gb|AAK29088.1|	CT	602
gi|15487369|gb|AAK50545.1|	CT	602
gi|15487379|gb|AAK50554.1|	CT	602
gi|20152868|gb|AAM13406.1|	CT	602
gi|40644703|emb|CAD92351.1|	CT	602
gi|40644710|emb|CAD92357.1|	CT	602
gi|40644717|emb|CAD92363.1|	CT	602
gi|13559814|ref|NP_112027.1|	CT	602
gi|57282813|emb|CAF25497.1|	CT	602
gi|307557085|gb|ADN51995.1|	CT	602
gi|313476293|emb|CBM41034.1|	CT	602
gi|66271898|gb|AAY43916.1|	CT	602
gi|374256950|gb|AEZ01382.1|	CT	602
gi|374256958|gb|AEZ01389.1|	CT	602
gi|253559848|gb|ACT32615.1|	CT	602
gi|374256967|gb|AEZ01397.1|	CT	602
gi|357640965|gb|AET87354.1|	--	318

Sequence logo 4.3bits 0.0bits 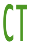

Supplement: Report S3 — Alignment of G proteins in all reported Nipah virus isolates in Genebank. (PDF) [file ppat.1003684.s010.pdf]
