# Supplementary material for: Crystal Structure of the Hendra Virus Attachment G Glycoprotein Bound to a Potent Cross-Reactive Neutralizing Human Monoclonal Antibody
Source: PLoS Pathog. 2013 Oct 10;9(10):e1003684. doi: 10.1371/journal.ppat.1003684 (PMC3795035; doi:10.1371/journal.ppat.1003684)
Supplement: Table S1 — Crystallographic data and refinement statistics. (DOC) [file ppat.1003684.s011.doc]

**Table S1. Crystallographic data and refinement statistics**

| m102.3/HeV-G | 1^st^ crystal form | 2^nd^ crystal form |
| --- | --- | --- |
| Space group | P6_1_22 | I222 |
| ASU content | 1 complex | 1 complex |
| Cell dimensions | a=b=72.871 Å, c=653.393  α=β=90°, γ=120° | a= 89.276Å, b=148.534Å, c=185.486Å  α=β=γ=90° |
| Resolution (Å) | 50-2.7 (2.75-2.7) | 50-2.8 (2.9-2.8) |
| Wavelength (Å) | 0.9792 | 0.9792 |
| Completeness (%) | 99.9 (100) | 99.9 (100) |
| Redundancy | 7.4 (7.6) | 7.3 (7.5) |
| I/σI | 17.0 (2.1) | 24.3 (2.8) |
| R_sym_ (%)* | 12.5 (71.4) | 8.7 (63.7) |
| R_free_/R (%)§ | 27.2 / 21.2 (32.2 / 25.2) | 25.2 / 19.4 (35.0 / 25.8) |
| No. atoms | 6910 | 6931 |
| r.m.s.d. bond (Å) | 0.008 | 0.009 |
| r.m.s.d. angle (°) | 1.318 | 1.563 |
| AVG B factor (Å^2^) | 56.2 | 63.2 |
| Fab | 72.8 | 65.9 |
| HeV-G | 41.3 | 60.8 |
| Ramachandran statistics |  |  |
| Favored: | 94.6% | 95.1% |
| Allowed: | 5.4% | 4.9% |

* *R*_sym_ =∑| *I* -<*I*>|/∑*I*, where *I* is the observed intensity and <*I*> is the statistically weighted absolute intensity of multiple measurements of symmetry related reflections.

§ *R* = ∑|*F*_o_ - *k*|*F*_c_||/∑|*F*_o_|, *R* from the working set and *R*_free_ from the test set.

The values in brackets are for the highest resolution shell.
