# Supplementary material for: Crystal Structure of the Hendra Virus Attachment G Glycoprotein Bound to a Potent Cross-Reactive Neutralizing Human Monoclonal Antibody
Source: PLoS Pathog. 2013 Oct 10;9(10):e1003684. doi: 10.1371/journal.ppat.1003684 (PMC3795035; doi:10.1371/journal.ppat.1003684)
Supplement: Table S2 — Affinity measurements of the mAb/G and ephrin-B2/G interactions performed using BioLayer Interferometry. EFNb2 is ephrin-B2. A bar graph of the measured KD values is also provided. (DOC) [file ppat.1003684.s012.doc]

**Table S2:** **Affinity measurement with BioLayer Interferometry**

|  |  | **K_D*_ (M)** | **k_on_ (1/Ms)** | **k_on_ error** | **k_off_ (1/s)** | **k_off_ error** |
| --- | --- | --- | --- | --- | --- | --- |
| **m102.3** | **NiVsG** | 5.56E-09 | 2.75E+05 | 7.04E+03 | 1.53E-03 | 7.13E-05 |
|  | **NiVsG(V507I)** | 4.21E-09 | 2.47E+05 | 4.48E+03 | 1.04E-03 | 4.88E-05 |
|  | **HeVsG** | 2.74E-08 | 4.57E+04 | 6.51E+02 | 1.25E-03 | 4.45E-05 |
|  | **HeVsG(D582N)** | 6.32E-08 | 2.64E+04 | 6.31E+02 | 1.67E-03 | 6.50E-05 |
|  |  |  |  |  |  |  |
| **m102.4** | **NiVsG** | 2.55E-08 | 1.37E+05 | 3.09E+03 | 3.49E-03 | 1.25E-04 |
|  | **NiVsG(V507I)** | 7.31E-09 | 1.61E+05 | 3.56E+03 | 1.18E-03 | 6.81E-05 |
|  | **HeVsG** | 1.11E-07 | 2.75E+04 | 6.84E+02 | 3.05E-03 | 9.48E-05 |
|  | **HeVsG(D582N)** | 3.00E-07 | 1.43E+04 | 4.50E+02 | 4.29E-03 | 1.37E-04 |
|  |  |  |  |  |  |  |
| **EFNb2** | **NiVsG** | 8.88E-09 | 1.66E+05 | 2.24E+03 | 1.48E-03 | 4.37E-05 |
|  | **NiVsG(V507I)** | 3.62E-09 | 1.59E+05 | 2.05E+03 | 5.76E-04 | 3.61E-05 |
|  | **HeVsG** | 1.73E-08 | 7.22E+04 | 9.04E+02 | 1.25E-03 | 3.73E-05 |
|  | **HeVsG(D582N)** | 2.36E-08 | 4.42E+04 | 5.15E+02 | 1.04E-03 | 3.28E-05 |

*note: K_D =_ k_off /_ k_on_

**Chart of K_D_ measurement:**
